# DNA methylome and single-cell transcriptome analyses reveal CDA as a potential druggable target for ALK inhibitor–resistant lung cancer therapy

**DOI:** 10.1038/s12276-022-00836-7

**Published:** 2022-08-23

**Authors:** Haejeong Heo, Jong-Hwan Kim, Hyun Jung Lim, Jeong-Hwan Kim, Miso Kim, Jaemoon Koh, Joo-Young Im, Bo-Kyung Kim, Misun Won, Ji-Hwan Park, Yang-Ji Shin, Mi Ran Yun, Byoung Chul Cho, Yong Sung Kim, Seon-Young Kim, Mirang Kim

**Affiliations:** 1 Personalized Genomic Medicine Research Center, Korea Research Institute of Bioscience and Biotechnology (KRIBB), Daejeon, 34141 Republic of Korea; 2 Department of Functional Genomics, KRIBB School of Bioscience, Korea University of Science and Technology (UST), Daejeon, 34113 Republic of Korea; 3 Korea Bioinformation Center, KRIBB, Daejeon, 34141 Republic of Korea; 4 Department of Internal Medicine, Seoul National University Hospital, Seoul, 03080 Republic of Korea; 5 Department of Pathology, Seoul National University College of Medicine, Seoul, 03080 Republic of Korea; 6 Department of Internal Medicine, Division of Medical Oncology, Yonsei Cancer Center, Yonsei University College of Medicine, Seoul, 03722 Republic of Korea; 7 Functional Genomics Institute, PDXen Biosystems Co., Daejeon, 34129 Republic of Korea

**Keywords:** Gene expression, Epigenomics, Non-small-cell lung cancer

## Abstract

Acquired resistance to inhibitors of anaplastic lymphoma kinase (ALK) is a major clinical challenge for *ALK* fusion-positive non-small-cell lung cancer (NSCLC). In the absence of secondary *ALK* mutations, epigenetic reprogramming is one of the main mechanisms of drug resistance, as it leads to phenotype switching that occurs during the epithelial-to-mesenchymal transition (EMT). Although drug-induced epigenetic reprogramming is believed to alter the sensitivity of cancer cells to anticancer treatments, there is still much to learn about overcoming drug resistance. In this study, we used an in vitro model of ceritinib-resistant NSCLC and employed genome-wide DNA methylation analysis in combination with single-cell (sc) RNA-seq to identify cytidine deaminase (CDA), a pyrimidine salvage pathway enzyme, as a candidate drug target. *CDA* was hypomethylated and upregulated in ceritinib-resistant cells. CDA-overexpressing cells were rarely but definitively detected in the naïve cell population by scRNA-seq, and their abundance was increased in the acquired-resistance population. Knockdown of CDA had antiproliferative effects on resistant cells and reversed the EMT phenotype. Treatment with epigenome-related nucleosides such as 5-formyl-2′-deoxycytidine selectively ablated CDA-overexpressing resistant cells via accumulation of DNA damage. Collectively, our data suggest that targeting CDA metabolism using epigenome-related nucleosides represents a potential new therapeutic strategy for overcoming ALK inhibitor resistance in NSCLC.

## Cancer: enzyme target to overcome drug resistance

A certain type of drug-resistant lung cancer may be susceptible to therapeutics directed against an enzyme involved in recycling nucleic acids. A team led by Mirang Kim and Seon-Young Kim from the Korea Research Institute of Bioscience and Biotechnology in Daejeon, South Korea, analyzed non-small-cell lung cancers that contain modified versions of a gene called ALK and have acquired resistance to a leading drug that inhibits the ALK protein. The researchers used single-cell analyses of RNA expression levels and genome-wide DNA methylation analysis to identify CDA, a gene that encodes the cytidine deaminase enzyme, as a mediator of drug resistance. They showed that therapeutically targeting the metabolism of this key enzyme led to the selective depletion of *CDA*-expressing cancer cells, a strategy that, if successful clinically, could help patients to overcome resistance to ALK-inhibiting drugs.

## Introduction

Anaplastic lymphoma kinase (ALK) is a receptor tyrosine kinase that is expressed in the nervous system, testes, and small intestine in adult humans^1^. As documented thus far, chromosomal rearrangements of *ALK* result in fusions with more than 20 different genes, and ALK fusion proteins drive tumorigenesis in many different cancers^2^. A fusion between *EML4* (echinoderm microtubule-associated protein-like 4) and *ALK* was identified in non-small-cell lung cancer (NSCLC) in 2007^3^. Since then, *ALK* fusions have been detected in ~3–7% of patients with NSCLC and have been associated with nonsmoking and younger age among patients^4^.

ALK inhibitors are highly effective at treating patients with *ALK* fusion-positive NSCLC, but the inevitable emergence of chemotherapeutic drug resistance limits their utility^2^. Genetic mechanisms such as secondary mutations in the ALK kinase domain explain the drug resistance observed in 20 and 50% of patients with first-generation (crizotinib) and second-generation (ceritinib and alectinib) treatment, respectively^5^. This suggests that nongenetic mechanisms contribute to drug resistance by modulating transcriptome plasticity^6^. It was recently suggested that drug-induced epigenetic reprogramming can alter the sensitivity of cancer cells to anticancer treatments^7^. Indeed, epigenome characteristics such as DNA methylation, histone modifications, and noncoding RNAs contribute to gene expression changes for adaptation in response to anticancer therapies^8^, but there is still much to learn about how epigenetic mechanisms contribute to drug resistance.

Acquisition of nucleotides is critical for DNA replication in proliferating cells. Nucleotides are produced either by de novo synthesis or by salvage pathways that recycle nucleobases from intracellular degradation processes or are acquired via extracellular uptake^9^. Cytidine deaminase (CDA) is an enzyme of the pyrimidine salvage pathway that catalyzes the deamination of cytidine and deoxycytidine to produce uridine and deoxyuridine, respectively^10^. Epigenome-related nucleosides such as 5-hydroxymethyl-2′-deoxycytidine (5hmdC) and 5-formyl-2′-deoxycytidine (5fdC) are produced by ten-eleven translocation (TET) methylcytosine dioxygenases^11^. Most cells can scavenge epigenome-related nucleosides and maintain genomic integrity, but cancer cells overexpressing CDA convert 5hmdC and 5fdC into variants of uridine, which accumulate in DNA and result in DNA damage and even cell death^12^. Therefore, epigenome-related nucleosides may present a new strategy for targeting tumors overexpressing CDA^13^.

We previously reported that enhancer remodeling and microRNA alterations can drive the epithelial-to-mesenchymal transition (EMT) and promote chemotherapeutic resistance to ALK inhibitors^14^. In the present study, we examined genome-wide changes in DNA methylation in acquired resistance to the anticancer drug ceritinib and identified *CDA* among the hypomethylated and upregulated genes in drug-resistant cells. Single-cell RNA sequencing (scRNA-seq) revealed rare CDA-overexpressing cells in the naïve cell population (those without acquired resistance) and that CDA-overexpressing cells with acquired resistance thrive when exposed to ceritinib. Finally, we found that treatment with epigenome-related nucleosides such as 5fdC are a promising therapeutic strategy for overcoming ALK inhibitor resistance in CDA-overexpressing NSCLC.

## Materials and methods

### Chemicals

Ceritinib (a.k.a., LDK378; S7083, Selleckchem, TX), 5hmdC (PY7588, Berry & Associates, MI), and 5fdC (PY7589, Berry & Associates) were dissolved in dimethyl sulfoxide (DMSO; D2650, Sigma–Aldrich, MO). Tetrahydrouridine (584222, Merck Millipore, NJ) was dissolved in water.

### Cell lines

The *EML4-ALK*-positive NSCLC cell Line H3122 was obtained as previously described in ref. ^14^ and cultured in RPMI-1640 medium (LM 011-01, Welgene, Gyeongsan, Korea) supplemented with 1% antibiotics (15240062, Gibco, NY) and 10% fetal bovine serum (S001-07, Welgene). Ceritinib (LDK378)-resistant (LR) cells were established previously^14^. Briefly, H3122 cells were cultured with increasing concentrations of ceritinib starting at the IC_30_ value. Resistant cells were obtained after ~6 months of culture with continuous 1 μM ceritinib treatment. All cells were maintained at 37 °C in a humidified atmosphere containing 5% CO_2_.

### Bulk RNA-seq analysis

Bulk RNA-seq was performed as described in ref. ^14^. The RNA-seq library was prepared using the TruSeq RNA Sample Prep kit (Illumina, CA), and sequencing was performed using the Illumina HiSeq2000 platform to generate 100-bp paired-end reads. The sequence reads were then mapped to the human genome (hg19) using STAR (v.2.5.1)^15^, and gene expression was quantified using the count module in STAR. The edgeR package (v.3.12.1)^16^ was employed to select differentially expressed genes (DEGs) from RNA-seq count data. The “trimmed mean of M-values” normalized value for each gene (in counts per million, cpm) was set at 1 and Log_2_-transformed for further analysis.

Quantitative reverse transcription-PCR (qRT–PCR) RNA was isolated using the RNeasy Plus Mini kit (74136, Qiagen, CA) and assessed using a NanoDrop ND-1000 spectrophotometer (Agilent, CA). cDNA was synthesized from 1 μg RNA using the iScript cDNA Synthesis kit (1708890, Bio-Rad, CA). Real-time PCR was conducted in triplicate for each sample according to the manufacturer’s instructions (170-8880AP, Bio-Rad). The value for each gene was normalized to the β-actin signal. Supplementary Table 1 lists the sequences of the primers used.

DNA methylation microarray analysis Genome-wide DNA methylation was analyzed in duplicate using the Infinium methylation 450 K beadchip array (Illumina), and the resulting DNA methylated/unmethylated signal intensity data were imported into R (v.3.4.2) for analysis. The data were normalized using the subset-quantile within array normalization method with background correction with the minfi (v.1.30.0) R package. CpG methylation values were calculated as average β values. Measurements with *P* < 0.05 were considered significant above background. To identify differentially methylated CpGs between H3122 and LR cells, statistical analysis was performed using the DMRcate 4 R package (v.1.20.0). Differentially methylated CpGs with *P* < 0.05 and an average difference >15% were selected.

### Preparation and sequencing of the scRNA-seq library

Samples were prepared as outlined in the 10× Genomics Single Cell 3′ v2 Reagent kit user guide. The single-cell RNA-seq (scRNA-seq) library was prepared using the Chromium Single Cell 3′ Library and Gel Bead kit V2 (PN-120237), Chromium Single Cell 3′ Chip kit V2 (PN-120236), and Chromium i7 Multiplex kit (PN-120262) with the 10× Genomics Chromium instrument. Samples were sequenced using a HiSeq 2500 with the following run parameters: read 1, 26 cycles; read 2, 98 cycles; index 1, 8 cycles. A median sequencing depth of 60,000 reads/cell was targeted for each sample. Supplementary Table 2 lists the 10× Genomics web summaries for each sample profiled.

### Bisulfite sequencing

Genomic DNA was isolated using the DNeasy Blood and Tissue kit (69506, Qiagen). Unmethylated cytosines were converted to uracil by sodium bisulfite using the EZ DNA Methylation-Gold kit (D5005, Zymo Research, CA). Modified DNA was amplified with primers targeting the CpG sites (cg04087271, cg20619374, and cg06984156) of *CDA* (Supplementary Table 3). Gel-purified bands were extracted using a Gel Extraction kit (28706, Qiagen) and cloned into the pGEM-T Easy vector (A1360, Promega, WI). Multiple plasmid DNA was isolated using the HTS Plasmid kit (PHTS-30, Core Bio System, Korea), and Sanger DNA sequencing was performed by GenoTech (Daejeon, Korea).

Western blot analysis Western blotting was performed as described^14^. Antibodies were diluted in 5% skim milk or 5% bovine serum albumin in Tris-buffered saline containing 0.1% Tween-20. The antibodies and dilutions used were as follows: anti-CDA (SAB1300717, Sigma–Aldrich, 1:500), anti-E-cadherin (1:1000, 3195, CST, MA), anti-N-cadherin (1:1000, 4061, CST), anti-vimentin (1:1000, 5741, CST), anti-GAPDH (1:2000, sc-47724, Santa Cruz Biotechnology, CA; 1:2000, 5174, CST), goat anti-mouse IgG (1:5000, 31430, Invitrogen, CA), and goat anti-rabbit IgG (1:5000, 31460, Invitrogen). Immunopositive bands were detected using an ECL kit (K-12045-D50, K-12042-D10, Advansta, CA) and visualized using a Fujifilm LAS-4000 (Tokyo, Japan).

### siRNA-mediated gene knockdown

Small interfering RNA (siRNA)-based gene knockdown was performed using Lipofectamine RNAiMAX 2000 (13778150, Invitrogen). Predesigned siRNAs were purchased from Bioneer along with nontargeting siRNA (SN-1002, Bioneer, Daejeon, Korea) as a control. Supplementary Table 4 lists these siRNA sequences.

### Preparation and sequencing of the scATAC-seq library

Samples were prepared as outlined in the 10× Genomics Single Cell ATAC Reagent kit v1.1 user guide. A single-cell assay of the transposase-accessible chromatin (scATAC) library was prepared using the Chromium Next GEM Single Cell ATAC Library and Gel Bead kit v1.1 (PN-1000175), Chromium Next GEM Chip H Single Cell kit v1.1 (PN-1000161), and Single Index kit (PN-1000212) with the 10× Genomics Chromium instrument. Samples were sequenced using a HiSeq 2500 with the following run parameters: read 1, 50 cycles; read 2, 50 cycles; index 1, 8 cycles; index 2, 16 cycles. A median sequencing depth of 25,000 reads/nucleus was targeted. The 10× Genomics web summary can be found in Supplementary Table 5.

### In vivo tumorigenicity assay

All animal experiments were conducted as approved by the Committee on Animal Experimentation of the Korea Research Institute of Bioscience & Biotechnology (KRIBB, Daejeon, Korea). Four-week-old female BALB/c nude mice were purchased from Orient Bio. LR cells (5 × 10^6^) in a Matrigel suspension (354248, Corning) were subcutaneously injected into the flanks of 5-week-old mice. Tumor size was measured using Vernier calipers every 3 days. Tumor volume was calculated as volume (mm^3^) = 0.532 × length × width^2^. When the tumor size reached 100–150 mm^3^, the mice were randomly divided into control and 5fdC groups. 5fdC was prepared in 20% polyethylene glycol 400 (807485, Sigma–Aldrich) and 3% Tween 80 (822187, Sigma–Aldrich) and administered at 100 mg/kg every 24 h for 13 days. Control mice were treated on the same schedule as vehicle. For immunofluorescence, 3-μm-thick mouse tissue samples were incubated with anti-Ki-67 (1:100, AB9260, Merck Millipore) and anti-γ-H2AX (1:100, 05-636, Merck Millipore) antibodies. The slides were then incubated with the secondary antibodies Alexa 488 and Alexa 647 (1:200, ab150077, ab150115, Abcam, Cambridge, UK) and 4′,6-diamidino-2-phenylindole dihydrochloride (DAPI; H-1800-2, Vector Laboratories, CA). Images were captured using an LSM 800 (ZEISS).

### Immunohistochemistry

Authorization for the use of paraffin-embedded human lung cancer samples for research purposes and ethical approval were obtained from the Institutional Review Board of Seoul National University Hospital (Seoul, Korea). Tissue sections for immunohistochemistry were cut at a 4-μm thickness from paraffin blocks of patient samples. The sections were stained with anti-CDA (ab222515, Abcam) using a Benchmark XT autostainer (Ventana Medical Systems, AZ). CDA expression was determined using the tumor proportion score, which is the percentage of viable tumor cells showing cytoplasmic staining. All samples were independently reviewed by a pathologist (J.K.) in a blinded manner.

### Statistical analysis

The number of biological replicates (*n*) is described in the figure legends. Sample size determination based on statistics was not applied in this study. Statistical analysis was performed using R software (v. 3.6.3). Data are presented as the mean ± SEM or SD, as indicated in figure legends. The researchers involved in this study were not completely blinded to the animal experiments but were blinded to the human data analyses. Statistical significance between the two groups was evaluated using Student’s *t*-test or the Mann–Whitney *U*-test, as appropriate. A *P* value of <0.05 was considered significant.

## Results

### Changes in the DNA methylome are associated with acquired resistance to ceritinib

**Fig. 1:**
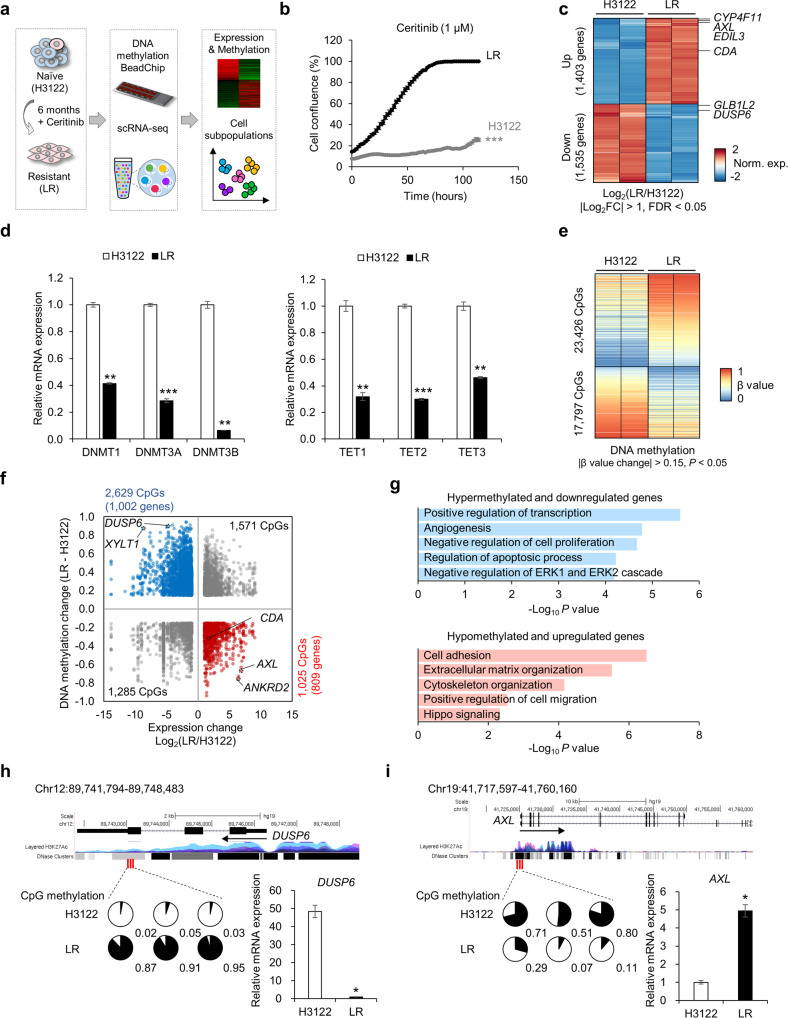
DNA methylome and transcriptome changes in ceritinib resistance. **a** Schematic of the experimental design. LR cells were established as described in ref. ^14^. DNA methylome and transcriptome changes were analyzed using DNA methylation 450k BeadChip, RNA-seq, and scRNA-seq. **b** Proliferation of H3122 and LR cells treated with 1 µM ceritinib using live-cell imaging. *n* = 3 independent experiments, mean ± SEM, ***P < 0.001 (unpaired two-sided *t*-test). **c** Heatmap showing DEGs in H3122 and LR cells. Filtering was based on cutoff values of |Log_2_Fold Change| >1.0 and FDR < 0.05. Norm. exp. normalized expression. **d** Relative mRNA expression of DNMTs and TETs, as analyzed by qRT–PCR. *n* = 3 independent experiments, mea*n* ± SD, **P < 0.01, ***P < 0.001 (Mann–Whitney *U*-test). **e** Heatmap of differentially methylated CpGs between H3122 and LR cells. Differential methylation was based on a *P* value < 0.05 and β value difference of 0.15. **f** Scatter plot showing the correlation between changes in DNA methylation and mRNA expression in H3122 and LR cells. Blue indicates CpGs from hypermethylated and downregulated genes (*n* = 1002); red indicates CpGs from hypomethylated and upregulated genes (*n* = 809). **g** Gene Ontology enrichment analysis of DEGs involved in biological processes. Terms are sorted by –Log_10_(*P* value). **h**, **i** Top: Maps of *DUSP6* (**h**) and *AXL* (**i**) from the UCSC Genome Browser. Bottom left**:** DNA methylation of *DUSP6* and *AXL* in H3122 and LR plotted as pie charts representing the percentage of methylation (black) of individual Infinium Human Methylation 450 K BeadChip probes (red lines): cg01814191, cg17740822, and cg05769889 for *DUSP6*; cg10564498, cg03247049, and cg12722469 for *AXL*. Total methylation ratios are indicated under each pie chart. Bottom right: Relative mRNA levels of *DUSP6* and *AXL* in H3122 and LR cells. *n* = 3 independent experiments, mean ± SD, *P < 0.05 (Mann–Whitney *U*-test).

We previously established an in vitro model of ceritinib-resistant NSCLC and confirmed that the model had no genetic aspects that could potentially confound analysis, such as secondary mutations in the ALK tyrosine kinase domain^14^. To explore changes in the DNA methylome and transcriptome during the development of acquired resistance to an ALK inhibitor in NSCLC, we performed 450 K BeadChip DNA methylation analysis and scRNA-seq of the *EML4-ALK* fusion-positive lung cancer cell line H3122 and the ceritinib-resistant cell line LR established (Fig. 1a, b). Previous bulk RNA-seq revealed dramatic differences in global gene expression between H3122 and LR cells (Fig. 1c)^14^. Among the genes detected by RNA-seq (*n* = 10,519), 13% (*n* = 1403) were upregulated and 14% (*n* = 1535) downregulated in LR cells (fold change (FC) >2, false-discovery rate (FDR) <0.05; Fig. 1c). *CYP4F11* (cytochrome P450 4F11), *EDIL3* (EGF-like repeats and discoidin domains 3), and *AXL* (AXL receptor tyrosine kinase) were the most strongly upregulated genes, whereas *DUSP6* (dual-specificity phosphatase 6) and *GLB1L2* (galactosidase beta 1-like 2) were the most strongly downregulated genes. DNA methyltransferases and TETs were also downregulated in LR cells (Fig. 1d), suggesting that acquired resistance may involve changes in not only the transcriptome but also the DNA methylome.

To determine whether the transcriptional changes observed are associated with DNA methylation changes, we analyzed genome-wide methylation changes using the Infinium Human Methylation 450 K array. A total of 23,426 CpGs were hypermethylated and 17,797 CpGs hypomethylated in LR cells (|β value change| > 0.15, *P* < 0.05; Fig. 1e). Integrated analyses of DNA methylation and gene expression revealed 1,002 genes, including *XYLT1* (xylosyltransferase 1) and *DUSP6*, to be hypermethylated and downregulated, and 809 genes, including *ANKRD2* (ankyrin repeat domain 2) and *AXL*, to be hypomethylated and upregulated (Fig. 1f). The hypermethylated and downregulated genes are associated with apoptosis, cell proliferation, and MAPK (mitogen-activated protein kinase) signaling; the hypomethylated and upregulated genes are associated with cell adhesion, cell migration, and Hippo signaling (Fig. 1g). These data suggest that transcriptional plasticity may drive stable epigenetic changes such as DNA methylation during the development of ceritinib resistance.

To examine differentially methylated and expressed genes more closely, we first focused on *DUSP6*. Reactivation of MAPK signaling is a hallmark of acquired resistance to ALK inhibitors in NSCLC^17^, and decreased expression of DUSP6, a MAPK phosphatase, promotes resistance to ALK inhibitors. Interestingly, CpG sites in exon 3 of *DUSP6*, but not the promoter region, were heavily methylated in LR cells (Fig. 1h), suggesting that these sites may be critical regulatory regions for *DUSP6* transcription. Other negative regulators of MAPK signaling, including *SPRY1*, *DAB2IP*, *ARRB1*, *DMD*, *CNKSR3*, *PTPRR*, *NLRP12*, *WNK2*, *SLC9A3R1*, *ERRFI1*, and *SPRY4*, were also hypermethylated and downregulated in LR cells (Supplementary Fig. 1). AXL is a receptor tyrosine kinase associated with tumor cell proliferation, metastasis, EMT, and drug resistance^18^. We previously showed that AXL activation during EMT is a primary feature of acquired resistance to ALK inhibitors^14,19^. The *AXL* promoter region was demethylated in cells with acquired resistance (Fig. 1i). These data suggest that drug-induced changes in DNA methylation have a key role in converting a transient transcriptional state to a stable resistant state.

### scRNA-seq reveals CDA-overexpressing cells in both resistant and nonresistant cells

**Fig. 2:**
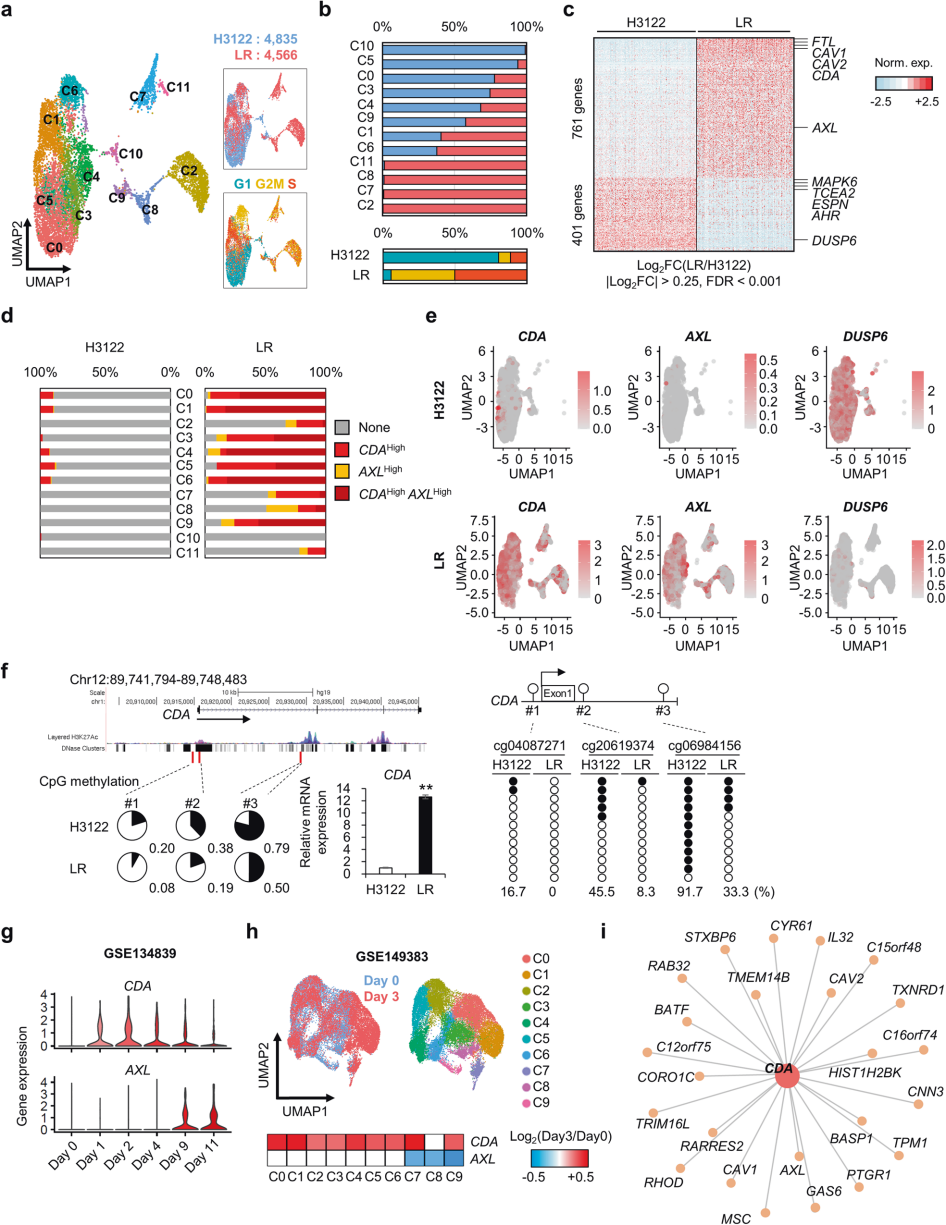
scRNA-seq analysis of H3122 and LR cells. **a** Left: Uniform manifold approximation and projection (UMAP) showing Clusters C0–C11 from H3122 and LR cells. Top right: UMAP plot showing scRNA-seq results for H3122 (*n* = 4835) and LR cells (*n* = 4566). Bottom right: UMAP plot representing scRNA-seq results based on cell cycle phases. **b** Top: Proportion of H3122 and LR cells in each cluster. Bottom: Proportion of H3122 and LR cells in each cell cycle phase. (Colors defined in Panel **a**) **c** Heatmap of DEGs between H3122 and LR cells. Filtering is based on cutoff values of |Log_2_FC| > 0.25 and FDR < 0.001. **d** Expression ratio of *CDA*^High^, *AXL*^High^, and *CDA*^High^ + *AXL*^High^ cells in each cluster for H3122 or LR cells. **e** Expression of *CDA*, *AXL*, and *DUSP6* assessed by UMAP. **f** Top: Map of *CDA* from the UCSC Genome Browser. Bottom left: DNA methylation of *CDA* in H3122 and LR cells. Pie charts represent the percentage of methylation (black) of the individual infinium human methylation 450 K BeadChip probes cg04087271, cg20619374, and cg06984156 (red lines). Total methylation ratios are indicated under each pie chart. Bottom right: Relative mRNA levels of *CDA* in H3122 and LR cells. *n* = 3 independent experiments, mean ± SD, **P < 0.01 (Mann–Whitney *U*-test). Far right: Bisulfite sequencing of *CDA* CpG sites (cg04087271, cg20619374, and cg06984156) in H3122 and LR cells. Closed and open circles represent methylated and unmethylated cytosines, respectively. The percentage of methylated cytosines is indicated at the bottom. **g** Violin plots showing *CDA* and *AXL* expression in PC9 cells treated with erlotinib for the indicated times. Data were obtained from GSE134839. **h** UMAP showing Clusters C0–C9 from PC9 cells treated for 3 days with erlotinib. Data were obtained from GSE149383. **i** Gene interaction map for interactions between *CDA* and related genes.

When attempting to identify tumor cell heterogeneity or driver cell populations, the traditional bulk RNA-seq method has limitations because it analyzes the gene expression profile of a mixture of cells. Recently, scRNA-seq technologies have allowed the investigation of RNA expression differences on a cell-by-cell basis^20^. To explore the heterogeneity of naïve and drug-resistant cells, we performed scRNA-seq using 10× Genomics Single Cell 3′ Solution and obtained gene expression profiles for 9401 cells. Clustering analysis of scRNA-seq data divided H3122 cells (*n* = 4835) and LR cells (*n* = 4566) into 12 clusters based on uniform manifold approximation and projection (Fig. 2a). Clusters 10 and 5 consisted mostly of H3122 cells, whereas Clusters 2, 7, 8, and 11 included mostly LR cells. Most LR cells were in the G2/M or S phase of the cell cycle (Fig. 2b and Supplementary Fig. 2a), whereas most H3122 cells were in G1, suggesting that LR cells are more proliferative than H3122 cells. Differential expression analysis with bulk RNA-seq data allows for the comparison of a limited number of biological replicates, but scRNA-seq can identify key players in subpopulations of cells^21^. We identified 761 upregulated genes and 401 downregulated genes in LR cells (|Log_2_FC| > 0.25, FDR < 0.001; Fig. 2c). EMT, cell cycle, and drug metabolism pathways were enriched in LR cells (Supplementary Fig. 2b); response to endoplasmic reticulum (ER) stress and cell adhesion molecules were enriched in H3122 cells (Supplementary Fig. 2c). *CDA* was one of the most significantly upregulated genes in LR cells (Fig. 2c). Intriguingly, although CDA-overexpressing cells were detected mainly among LR cells, they were also found rarely (yet definitively) in the naïve H3122 cell population (Fig. 2d, e). CDA is a nucleoside metabolism enzyme involved in the homeostasis of the cellular pyrimidine pool^22^. CDA-overexpressing cells in the naïve cell population showed enriched expression of cancer stem cell-related genes such as *S100A10* (S100 calcium-binding protein A10)^23^, *LGALS1* (galectin-1)^24^, and *SH3BGRL3* (SH3 domain-binding glutamate-rich protein-like 3)^25^ (Supplementary Table 6). These data show that CDA-overexpressing cells may possess a growth advantage during ceritinib treatment. *CDA* mRNA expression was increased 12-fold in LR cells, and methylation was reduced in the promoter and putative enhancer regions of *CDA* (Fig. 2f).

To investigate whether *CDA* expression is increased in acquired resistance to other tyrosine kinase inhibitors (TKIs), we analyzed bulk RNA-seq data for H3122 cells treated with crizotinib^26^. Consistent with our observations of ceritinib resistance, *CDA* and *AXL* were upregulated and *DUSP6* downregulated in crizotinib-resistant cells (Supplementary Fig. 2d). Furthermore, we analyzed scRNA-seq data for PC9 cells treated with erlotinib, an EGFR TKI, for 0, 1, 2, 4, 9, and 11 days^27^. Astonishingly, *CDA* was upregulated even on Day 1, whereas *AXL* was upregulated only after 9 days (Fig. 2g). On Day 3 of erlotinib treatment, *CDA* expression was increased in the overall clusters, whereas *AXL* expression was decreased compared with Day 0 (Fig. 2h). These data demonstrate that CDA-overexpressing cells may have a growth advantage during TKI treatment, causing them to be selected during treatment and thereby contributing to acquired resistance.

We identified 25 common genes that showed a high correlation (*R* > 0.3) with *CDA* expression in our scRNA-seq data and increased expression in both ceritinib-resistant (Fig. 1c) and crizotinib-resistant cells (Supplementary Fig. 2d, i). These included genes involved in EMT (*CAV1*, *CAV2*, *BASP1*, *C12orf75*, *GAS6*, *HIST1H2BK*, *IL32*, *CYR61*, *AXL*, *TRIM16L*, *TPM1*, *CORO1C*, *CNN3*, *RAB32*, and *RARRES2*), cell cycle regulation (*PTGR1*, *TMEM14B*, *RHOD*, and *C16orf74*), and nucleotide metabolism (*TXNRD1*), suggesting that CDA may have a key role in EMT as well as in the proliferation of cells with acquired resistance to ALK inhibitors.

### CDA depletion reverses EMT and reduces the proliferation and migration of cells with acquired resistance

**Fig. 3:**
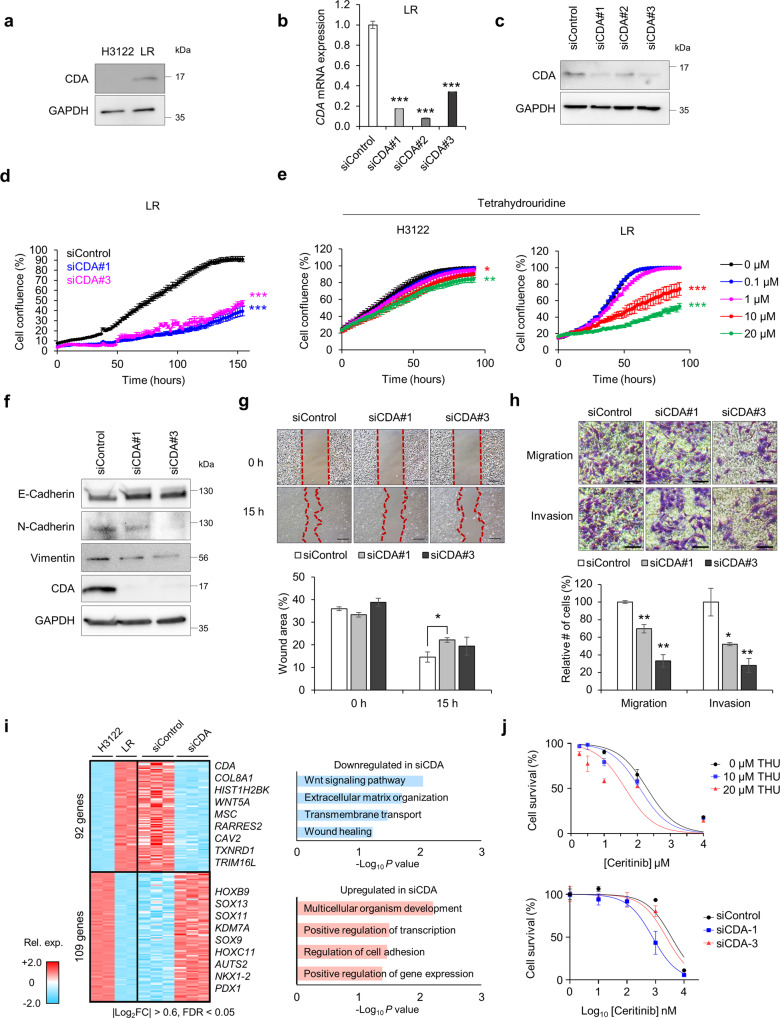
Effects of CDA knockdown on ceritinib-resistant cells. **a** Western blot analysis of CDA in H3122 and LR cell lysates. **b**, **c** Knockdown efficiency of siRNAs as assessed by qRT–PCR (**b**) and western blotting (**c**). *n* = 3 independent experiments, mean ± SD, ***P < 0.001 (Mann–Whitney *U*-test). For western blotting, GAPDH was used as a loading control. **d** Proliferation of LR cells transfected with siRNA for the indicated times. *n* = 3 indepe*n*dent experiments, mean ± SEM, ***P < 0.001 (unpaired two-sided *t*-test). **e** Proliferation of H3122 and LR cells treated with tetrahydrouridine (THU) (0–20 µM) for the indicated times. *n* = 3 independent experiments, mean ± SEM, *P < 0.05, **P < 0.01, ***P < 0.001 (unpaired two-sided *t*-test). **f** Expression of EMT-related proteins after CDA knockdown. GAPDH was used as a loading control. **g** Top: Wound healing analysis of CDA-depleted LR cells at 0 and 15 h after the cell surface was scratched. Scale bars, 100 µm. Bottom: Relative cell-covered wound area at 0 and 15 h. *n* = 3 independent experiments, mean ± SD, *P < 0.05 (Ma*n*n–Whitney *U*-test). **h** Top: Representative microscopic images of migrating and invading CDA-depleted LR cells 48 h after seeding. Scale bars, 100 µm. Bottom: Relative numbers of migrating and invading cells. *n* = 3 independent experiments, mean ± SD, *P < 0.05, **P < 0.01 (Mann–Whitney *U*-test). i Left: Heatmap showing the expression profiles of common DEGs between CDA knockdown (siControl vs. siCDA) and ceritinib resistance (H3122 vs. LR). Filtering was based on cutoff values of |Log_2_FC| > 0.6 and FDR < 0.05. Rel. exp. relative expression. Right: Gene Ontology enrichment analysis of DEGs involved in biological processes. Terms were sorted by –Log_10_ *P* value. **j** LR cells were treated with serially diluted ceritinib in the absence or presence of THU (top) or after CDA knockdown (bottom). Cell viability was determined using CCK-8. The number of viable cells was measured at 72 h. *n* = 3 independent experiments, mean ± SEM.

Western blotting confirmed an increased level of cellular CDA in resistant cells (Fig. 3a). To elucidate the function of CDA in acquired resistance, we depleted CDA in LR cells using three different CDA siRNAs. All three siRNAs reduced both CDA mRNA and protein levels (Fig. 3b, c). Notably, CDA depletion reduced LR cell proliferation (Fig. 3d). In addition, treatment with tetrahydrouridine (THU), a competitive inhibitor of CDA^28^, had a dose-dependent inhibitory effect on the proliferation of LR but not H3122 cells (Fig. 3e). Because CDA expression correlated with expression of EMT-related genes (Fig. 2i), we next examined whether CDA knockdown in LR was able to reverse the EMT phenotype. As expected, CDA depletion increased the expression of the epithelial marker E-cadherin and decreased that of the mesenchymal markers N-cadherin and vimentin (Fig. 3f). Furthermore, CDA depletion reduced wound healing (Fig. 3g) as well as cell migration and invasion (Fig. 3h).

Next, RNA-seq was used to investigate transcriptome changes caused by CDA depletion in LR cells (Fig. 3i). Intriguingly, 92 genes that were upregulated in LR cells were downregulated in CDA-depleted LR cells (|Log_2_FC| > 0.6, FDR < 0.05). These genes are related to EMT, extracellular matrix organization (*SMOC2*, *MMP13*, *ITGAX*, *SPP1*, and *COL8A1*), and transmembrane transport (*ATP8A1*, *ABCC5*, *SLCO2B1*, *ABCA4*, and *CYBRD1*). Five of the 25 previously identified CDA-linked genes (Fig. 2i) were downregulated by CDA silencing (*CAV2*, *HIST1H2BK*, *TXNRD1*, *TRIM16L*, and *MSC*); however, 109 genes that were downregulated in LR cells were significantly upregulated in CDA-depleted LR cells. Among those, transcription factors involved in the regulation of development and cell adhesion (*HOXB9*, *AUTS2*, *BCL11B*, *PDX1*, *SOX11*, *ARRB1*, and *SOX9*) were significantly upregulated by CDA depletion. These data suggest that CDA may drive LR cell transformation. We further assessed whether CDA inhibition can restore ceritinib sensitivity in LR cells. Indeed, the combination of ceritinib and THU more effectively inhibited LR cell growth than ceritinib alone (Fig. 3j top). Furthermore, CDA inhibition using siRNA increased ceritinib sensitivity (Fig. 3j bottom). Taken together, these data show that CDA may promote cell survival by increasing both cell proliferation and EMT in response to treatment with an ALK inhibitor.

### scATAC-seq reveals gene regulatory networks in cells with acquired resistance

**Fig. 4:**
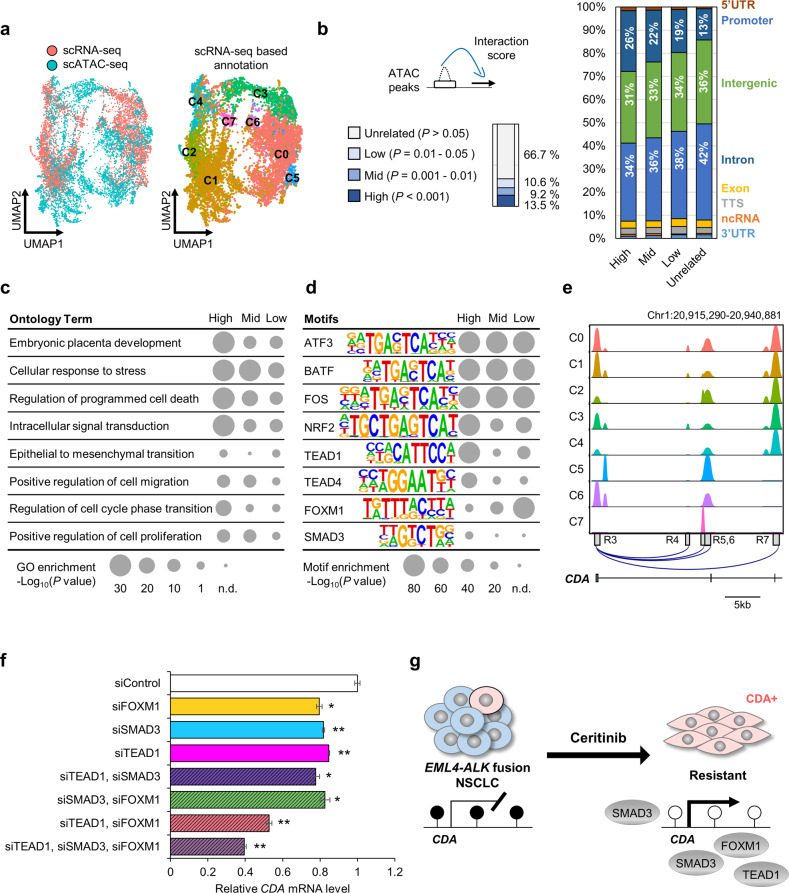
Integrated analysis of scRNA-seq and scATAC-seq in LR cells. **a** Left: Integrated scRNA-seq (*n* = 4566) and scATAC-seq (*n* = 6772) described by UMAP. Right: Annotated scATAC-seq clusters based on scRNA-seq. **b** Left: Calculation and classification of interaction scores. Right: Distribution of different genomic regions in the open chromatin region (OCR) groups. **c** Genomic region enrichment of annotations tool ontology analysis of OCRs. The *P* values shown are Bonferroni adjusted (n.d. not detected). **d** Transcription-factor motif enrichment analysis of OCRs. The most highly enriched motifs are shown. **e** ATAC signal for each cluster in the gene body of *CDA*. OCRs regulating *CDA* expression are displayed with the UCSC Genome Browser. **f** Relative *CDA* mRNA expression following knockdown of the indicated TFs using siRNAs. *n* = 3 independent experiments, mean ± SD, *P < 0.05, **P < 0.01 (Mann–Whitney *U*-test). **g** Schematic diagram of the regulatory roles of TEAD1, SMAD3, and FOXM1 in CDA activation in ceritinib-resistant cells.

The accessible chromatin landscape of resistant LR cells was established using scATAC-seq, which yielded profiles for 7753 nuclei, with a median of 6821 fragments mapped per nucleus (Supplementary Table 5). To identify *cis*-regulatory elements controlling gene expression in LR cells, we performed an integrated analysis of scRNA-seq with scATAC-seq. Based on scRNA-seq data, scATAC-seq data for each cluster were predicted with a gene activity score using SnapATAC software (Fig. 4a)^29^. To evaluate the relationship between open chromatin regions (OCRs) and gene expression, interaction scores were calculated by comparing the expression level of each gene with the chromatin accessibility of each OCR located within ±250 kb of the transcription start site (TSS). The OCRs were then categorized into four groups based on the interaction score: high, mid, low, and unrelated (Fig. 4b). The high group consisted of 13.5% of all OCRs and included more promoter regions than any other group. Gene Ontology analysis revealed that OCRs of LR cells are proximal to genes involved in cellular responses to stress, regulation of cell death, and cell migration (Fig. 4c). To dissect causal TFs responsible for the transcriptome profile of LR cells, we performed TF motif analysis of OCRs using HOMER^30^, revealing enrichment of TF-binding signatures such as the ATF3, BATF, and NRF2 sequence motifs (Fig. 4d).

With regard to *CDA* specifically, we identified 10 OCRs (R1-R10) within ±250 kb of the TSS significantly associated with *CDA* expression (Supplementary Fig. 3a, b). OCRs R3–R7 are located in the *CDA* gene body, and R6 displayed the most significant correlation with *CDA* expression (Fig. 4e and Supplementary Fig. 3c), suggesting that it may be a strong enhancer of *CDA*. Supplementary Fig. 3d, e lists the top TFs predicted to bind the 10 OCRs. Among the *CDA*-associated TFs (Supplementary Table 7), we focused on FOXM1, TEAD1, and SMAD3. FOXM1, a proliferation-specific TF, mediates EMT-associated EGFR TKI resistance^31^; it promotes rapid cancer cell proliferation in small-cell lung cancer and is associated with poor prognosis^32^. SMAD3 mediates transcriptional activation of EMT target genes in the TGFβ signaling pathway^33^. TEAD1 is a key TF in various oncogenic signaling pathways, including the Hippo, Wnt, TGFβ, and EGFR pathways, and plays critical roles in EMT, metastasis, drug resistance, and cancer stem cells^34^. To determine the effect of these TFs on the expression of *CDA*, we depleted the TFs using siRNAs and found that depletion of any one TF reduced *CDA* expression by ~20% and that depletion of a combination of two or three TFs had synergistic effects (Fig. 4f).

Taken together, these data indicate that an open chromatin structure may be formed in the promoter and enhancer regions of *CDA* at least in part via DNA demethylation in cells with acquired resistance. Furthermore, TFs such as TEAD1, SMAD3, and FOXM1 may be recruited to the regulatory region and induce overexpression of *CDA*, which promotes acquired resistance to ALK inhibitors (Fig. 4g).

### 5-Formyl-2′-deoxycytidine (5fdC) selectively ablates CDA-overexpressing cells

**Fig. 5:**
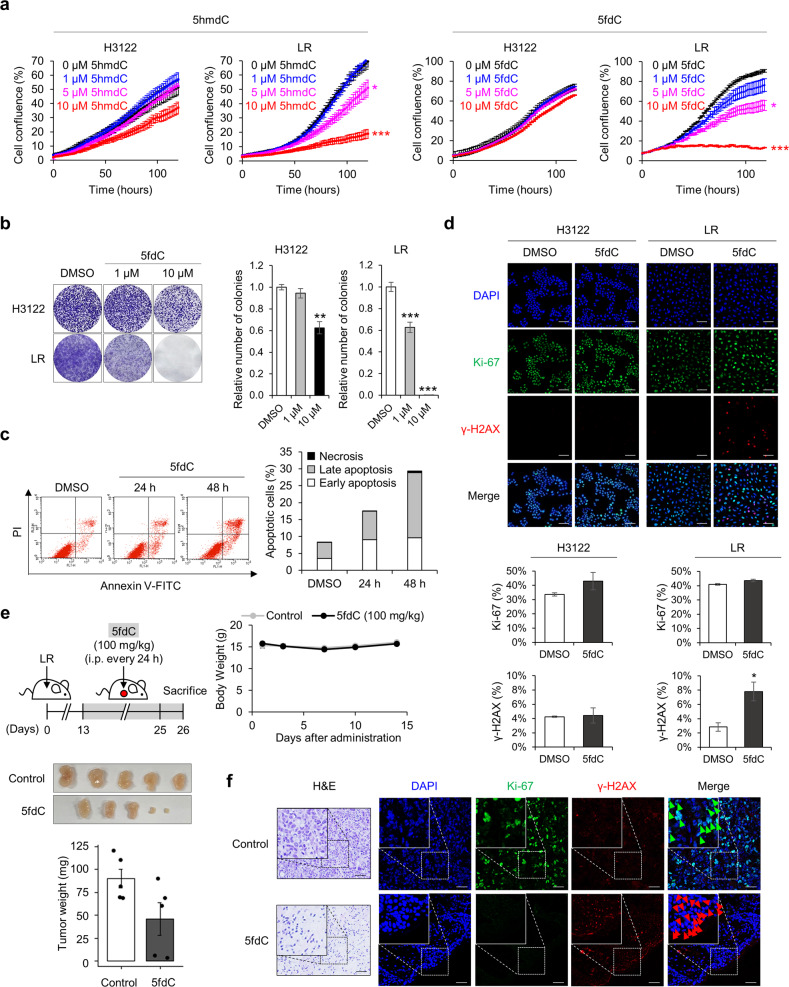
Antiproliferative effects of 5fdC on ceritinib-resistant cells. **a** Proliferation of H3122 and LR cells treated with 5hmdC (left) or 5fdC (right) for the indicated times. *n* = 3 independent experiments, mean ± SEM, *P < 0.05, ***P < 0.001 (unpaired two-sided *t*-test). **b** Colony forma*t*ion assay of H3122 and LR cells treated with DMSO (control) or 5fdC (1 or 10 µM) for 10 days. Left: Representative images. Right: Relative numbers of colonies. *n* = 3 independent experiments, mean ± SD, **P < 0.01, ***P < 0.001 (Mann–Whitney *U*-test). **c** Left: Flow cytometry analysis of annexin V and propidium iodide (PI) staining of LR cells treated with 5fdC (10 µM) for 24 or 48 h. Right: Histogram showing the percentages of cells in early apoptosis, late apoptosis, and necrosis. **d** Immunofluorescence labeling of Ki-67 (cell proliferation) and γ-H2AX (DNA damage) in H3122 and LR cells. Cells were treated with DMSO (control) or 5fdC (10 µM) for 24 h. Nuclear DNA was stained with DAPI (blue). Top: Representative images. Scale bar, 100 µm. Bottom: Fluorescence intensity of Ki-67 and γ-H2AX. *n* = 3 independent experiments, mean ± SEM, *P < 0.05 (Mann–Whitney *U*-test). **e** In vivo treatment of mice with LR cells and 5fdC. Top left: Schematic diagram of treatment. Top right: Mouse body weight over the course of 5fdC administration. Bottom: Photographs of dissected tumors. Bar plot of dissected tumor weight; mean ± SD (*n* = 5, Mann–Whitney *U*-test). **f** Evaluation of proliferation (Ki-67) and DNA damage (γ-H2AX) in dissected tumor samples. Representative images. Scale bar, 50 µm (H&E), 20 µm (Immunofluorescence).

CDA converts 5hmdC and 5fdC to 5hmdU and 5fdU, respectively, both of which induce cytotoxicity when incorporated into DNA. Therefore, cytidine variants such as 5hmdC and 5fdC have been suggested as drug treatments for CDA-overexpressing cancers^12^. We examined whether 5hmdC or 5fdC inhibits LR cell proliferation. As expected, treatment of LR cells with 5hmdC or 5fdC decreased proliferation in a dose-dependent manner. Notably, high doses of 5fdC (10 µM) specifically decreased the proliferation of LR cells but not H3122 cells, suggesting that it selectively inhibits CDA-overexpressing resistant cell proliferation (Fig. 5a). We then examined the effect of 5fdC on the survival of LR cells and found that 5fdC attenuated their colony-forming ability (Fig. 5b) and increased apoptosis (Fig. 5c). To determine whether 5fdC causes DNA damage specifically in LR cells, we carried out immunofluorescence staining for γ-H2AX, a marker of double-stranded DNA breaks. LR cells treated with 10 µM 5fdC showed a 2.7-fold increase in the number of cells with DNA damage (Fig. 5d). To investigate the effect of 5fdC on tumor growth, a xenograft assay was carried out using LR cells (Fig. 5e). Beginning on Day 13 after injection of LR cells, 5fdC (100 mg/kg) was administered each day by intraperitoneal injection. Compared with control mice, 5 fdC-treated mice did not show significant changes in body weight but did exhibit reductions in tumor weight (Fig. 5e). Furthermore, tumors in 5fdC-injected mice showed a decrease in the number of proliferating cells and an increase in the number of cells with DNA damage (Fig. 5f). These results demonstrate that CDA-overexpressing resistant cells are vulnerable to 5fdC owing to the accumulation of DNA damage and that 5fdC use is a potential strategy for overcoming ceritinib resistance.

### Clinical relevance of CDA in lung cancer patients

**Fig. 6:**
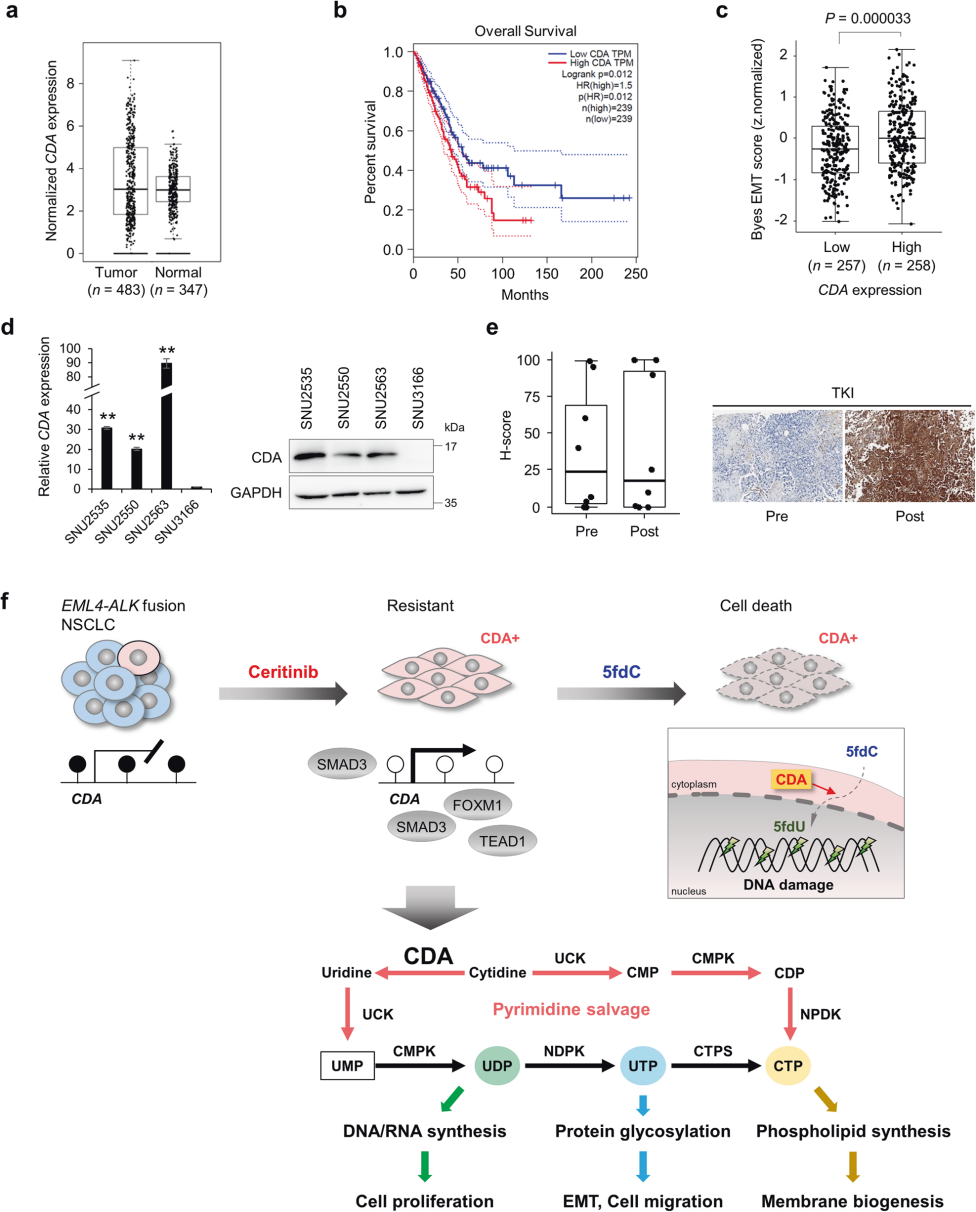
Clinical relevance of CDA expression in NSCLC patients. **a** *CDA* expression in lung adenocarcinoma tumor (*n* = 483, from TCGA) and normal (*n* = 347, from GTEx) tissue samples. Each dot represents an expression in a single sample. **b** Overall survival of lung adenocarcinoma patients with low or high *CDA* expression as analyzed by the Kaplan–Meier method and log-rank test. Median values in transcripts per million (TPM) are indicated by solid red and blue lines. The highest dotted red line is for a sample with higher expression than the median value for the high-expression cohort; the lowest dotted blue line is for a sample with lower expression than the median value for the low-expression cohort. HR hazard ratio. **c** EMT scores in lung adenocarcinoma tumor samples with low (*n* = 257) or high (*n* = 258) *CDA* expression. **d** Levels of *CDA* mRNA (left) and protein (right) assessed by qRT–PCR and western blotting of primary cancer cells from *ALK*-rearranged NSCLC patients with acquired resistance to crizotinib. *n* = 3 independent experiments, mean ± SD, **P < 0.01 (Mann–Whitney *U*-test). **e** Immunohistochemical staining of CDA in tumor biopsies from 11 NSCLC patients with *ALK* rearrangements before and/or after ALK inhibitor therapy. Left: Boxplot depicting CDA H-score distribution in NSCLC patients pretreated (*n* = 8) or posttreated (*n* = 8) with TKIs. Right: Representative images. **f** Model for targeting CDA-directed metabolism with 5fdC to overcome resistance to ALKis. CDA-overexpressing cells were preexisting in the naïve cell population and propagated during acquired resistance. Overexpression of this pyrimidine salvage pathway enzyme promotes cell proliferation, migration, and EMT. Epigenome-related nucleosides such as 5fdC can induce DNA damage through an accumulation of 5fdU in the DNA and ultimately lead to cell death in CDA-overexpressing resistant cells.

To explore the clinical relevance of CDA in lung cancer, we analyzed *CDA* expression in lung adenocarcinomas using GEPIA^35^, a tool for analyzing the Cancer Genome Atlas (TCGA) and Genotype-Tissue Expression (GTEx) databases. Although median *CDA* expression was similar between tumor samples (*n* = 483, obtained from TCGA) and normal tissue samples (*n* = 347, obtained from GTEx), ~25% of the tumor samples overexpressed *CDA* compared with normal samples (Fig. 6a). In addition, the overall survival of patients with lung adenocarcinoma with high *CDA* expression was significantly lower than that of patients with low *CDA* expression (Fig. 6b).

To determine whether *CDA* expression is associated with the EMT signature in lung adenocarcinoma, we investigated EMT scores in low and high *CDA* expression groups by surveying a 16-gene signature of canonical EMT from TCGA datasets^36^. Notably, the high *CDA* expression group had significantly higher EMT scores than the low group (Fig. 6c). Thus, CDA appears to have a key role in EMT during lung cancer progression.

We further examined CDA expression in primary cancer cells from NSCLC patients with *ALK* rearrangement who exhibited acquired resistance to crizotinib^37^. Notably, CDA expression was higher in patient-derived resistant cells (SNU-2535, -2550, -2563) than in naïve *ALK*-positive NSCLC patient-derived cells (SNU-3166) at both mRNA and protein levels (Fig. 6d and Supplementary Table 8). We obtained NSCLC tumor biopsies from 11 patients with *EML4*-*ALK* rearrangements before and/or after ALK inhibitor therapy. Contrary to our expectation, immunohistochemistry of CDA showed no significant difference between pre- and post-ALK inhibitor therapy (Fig. 6e, left), which might be attributable to inter- or intratumoral heterogeneity of CDA expression even in the same patient^38^. Representative CDA expression in tumors from pre- or post-TKI therapy is shown in Fig. 6e.

## Discussion

Proliferating cancer cells undergo metabolic adaptations to survive in the harsh tumor microenvironment^39,40^. Therefore, targeting cancer-specific metabolism may be an effective therapeutic strategy^40,41^. Our current results demonstrate epigenetic activation of *CDA* during the development of ceritinib resistance in NSCLC with *EML4*-*ALK* fusion. scRNA-seq analysis identified CDA upregulation as one of the primary characteristics of ALK inhibitor resistance. CDA-overexpressing cells have a growth advantage during ceritinib treatment and are thus become selected and propagated, contributing to acquired resistance. Decreased methylation of the *CDA* promoter and enhancer, along with the recruitment of EMT-related TFs, can at least partially explain the increased expression of CDA in resistant cells. We propose that targeting CDA-directed metabolism with epigenome-related nucleosides such as 5fdC represents a new strategy for ablating ALK inhibitor-resistant cells via accumulation of DNA damage leading to cell death (Fig. 6f).

As de novo pyrimidine biosynthesis is an energetically expensive pathway for cell growth and development^42^, CDA may provide an energetically efficient bypass for rapidly proliferating LR cells via a salvage pathway using either intracellular nucleic acid degradation products or extracellular nucleosides^43^. Consistent with this expectation, LR cells showed 1.2-fold more rapid cell growth than H3122 cells (Fig. 5a) and were mainly in the G2 M or S phase of the cell cycle (Fig. 2b). We also found that inhibition of CDA using siRNA or THU reduced LR cell proliferation (Fig. 3). Many studies have shown that reprogramming of pyrimidine metabolism is closely related to cancer progression^44^. Beyond DNA and RNA biosynthesis, recycling of cytidine and uridine is involved in phospholipid synthesis for cell membrane biogenesis^45^ and protein glycosylation for cell–cell and cell–matrix adhesion^46^ (Fig. 6f). Furthermore, the pyrimidine salvage pathway is involved in the ER stress response and may increase the adaptive capacity of cells to drug treatment^47^.

CDA also has critical role in EMT. CDA knockdown in LR cells reversed EMT and reduced cell migration and invasion (Fig. 3). Clinically, CDA expression is associated with the EMT signature in lung cancer patients (Fig. 6c). EMT is a dynamic process in which tumor cells can occupy intermediate EMT states (partial EMT) and can revert to a more epithelial phenotype through the reverse process, i.e., the mesenchymal-to-epithelial transition. Epigenetic changes such as DNA methylation and histone modifications direct this dynamic process^48^. We found the promoter and enhancer regions of *CDA* to be demethylated in cells with acquired resistance, forming an open chromatin structure to bind TFs such as SMAD3, TEAD1, and FOXM1 (Fig. 4). CDA expression was also linked to EMT-related genes such as CAV2, TXNRD1, and HISTH2BK (Fig. 2i). EMT has been associated with both metastasis and drug resistance^49^.

CDA is frequently overexpressed in many cancers, including pancreatic, stomach, testicular, and vaginal cancer^50^. Moreover, CDA overexpression can mediate resistance to chemotherapy based on cytidine analogs such as gemcitabine^51^. Based on our findings using ceritinib-resistant cells, we searched public data to determine whether upregulation of CDA is associated with resistance to other anticancer drugs. Intriguingly, CDA upregulation has been found in cancer cells resistant to palbociclib (CDK 4/6 inhibitor)^52^, trametinib (MEK inhibitor)^53^, olaparib (PARP inhibitor)^54^, and gefitinib (EGFR inhibitor)^55^ (Supplementary Table 9). Therefore, it appears that CDA has the potential to promote cancer cell survival in the presence of anticancer drugs. Although much biological and clinical validation is needed, targeting CDA with THU or epigenome-related nucleosides may enhance the effectiveness of current strategies for overcoming resistance to these targeted therapies.

Modified nucleosides that are common in the epigenome can disrupt the regulation of gene expression if they are recycled and incorporated into DNA, and thus incorporation must be prevented in most healthy cells. In the case of 5-methyl-2′-deoxycytidine (5mdC), it can be recycled in a different form, i.e., deoxythymidine triphosphate (dTTP), through deamination^22^. In contrast, the oxidized epigenetic nucleosides 5hmdC and 5fdC cannot be converted to canonical nucleotides in normal cells because cytidine monophosphate kinase 1 (CMPK1) phosphorylates only unmodified dCMP^12^. In CDA-overexpressing cancer cells, however, 5hmdC and 5fdC can be deaminated to yield 5hmdU and 5fdU, respectively, which can be incorporated into DNA, leading to cell cycle arrest and/or accumulation of cytotoxic double-stranded DNA breaks that cause cell death^12^. We demonstrated that administration of 5fdC inhibits the proliferation of ALK inhibitor-resistant NSCLC cells in vitro and in vivo (Fig. 5). Although our in vivo study was carried out with a very small number of mice, 5fdC selectively abolished CDA-overexpressing cells with no adverse effects on the animals. Zauri et al. have demonstrated the safety of 5hmdC and 5fdC administration based on a lack of a change in behavior or body weight in mice^12^. They also showed a lack of histological side effects in CDA-expressing organs such as the kidney and intestine^12^ and suggested that the cytotoxic thresholds of 5hmdC and 5fdC would be reached only in highly proliferating and CDA-overexpressing cells. Nonetheless, more extensive studies are needed to fully establish the efficacy and safety of 5hmdC and 5fdC as drugs for the treatment of cancer.

Taken together, this study provides proof of concept that single-cell transcriptome analysis can identify key players in acquired resistance to cancer therapy and that metabolic mechanisms may provide a vulnerability in such cancer cells that can be utilized to overcome resistance to targeted therapies. In this study, we focused on CDA because CDA-overexpressing cells were present in the naïve cell population and were propagated during ceritinib treatment. Future studies will be required to dissect the details of the role played by CDA in the EMT and the survival of therapy-resistant cancer cells during targeted therapy.

## Supplementary information

Supplementary information

## Data Availability

RNA-seq data were deposited in the NCBI Gene Expression Omnibus (GEO) under accession number GSE81484. Raw sequence tags were deposited in the NCBI Short Read Archive under accession number SRP075253. scRNA-seq and DNA methylation data were deposited in GEO under accession number GSE139388. scATAC-seq data were deposited in GEO under accession number GSE139388. The raw data for this study were deposited in the Korean Nucleotide Archive (KoNA) under accession number PRJKA210059.
