# Supplementary material for: DNA methylome and single-cell transcriptome analyses reveal CDA as a potential druggable target for ALK inhibitor–resistant lung cancer therapy
Source: Exp Mol Med. 2022 Aug 23;54(8):1236–49. doi: 10.1038/s12276-022-00836-7 (PMC9440127; doi:10.1038/s12276-022-00836-7)
Supplement: Supplementary file 1 — Supplementary information [file 12276_2022_836_MOESM1_ESM.pdf]

SUPPLEMENTARY MATERIALS AND METHODS

Gene set and pathway analysis

Gene set and pathway analysis was performed on DEGs associated with differentially methylated regions using DAVID (v.6.8) and enrichR. A cut-off threshold of $P < 0.05$ was used to obtain significantly enriched pathways. Gene sets enriched with at least three genes were considered for further analysis.

scRNA-seq data processing and analysis

Data preprocessing was performed following the pipeline provided by 10× Genomics. The process of converting raw sequencing data (Base Call files) into FASTQ files and aligning to the human reference genome (hg19) was performed using 10× Genomics cellranger software. The Cell Ranger count module was used to generate the raw gene expression matrix, and the Cell Ranger aggr module was used to aggregate the H3122 and LR data. The rest of the process was performed using R (*https://www.R-project.org/*) and a guided analysis pipeline from Seurat 5, v.2.4[1].

Cell and gene filtering processes included only genes expressed in at least three cells and cells expressing at least 200 genes. Cells with a mitochondrial genome transcript ratio greater than 50% were considered lysed and were excluded. For data normalization, the global-scaling normalization method LogNormalize (Seurat 5, v.2.4) was used with default parameters. The selection of variable genes, scaling of data, and linear dimensional reduction were performed sequentially following the Seurat-guided analysis pipeline. To determine the dimensionality of the dataset, we analyzed the elbow plot and determined 10 principle components. Cells were clustered using the Seurat FindClusters module, and clusters were visualized using uniform manifold approximation and projection (UMAP). To find DEGs in each cluster, we used the FindAllMarkers module (Seurat 5, v.2.4).

Cell proliferation assay using the IncuCyte system

Cell proliferation rates were determined based on cell confluency by live-cell imaging using the IncuCyte ZOOM System (Essen Bioscience, MI). H3122 or LR cells were seeded in 96-well plates (3596, Corning, NY), and photomicrographs were taken at 2-h intervals from four separate regions

per well using a 10× objective. Cultures were maintained in a 37°C incubator. Cell confluency was measured using IncuCyte software (Essen Bioscience).

Wound healing assay

To assess wound healing, 2 × 10^5 cells were seeded in a 35-mm culture insert μ-Dish (81176, IBIDI, WI) and incubated overnight at 37°C. When cells were attached, the insert well was removed and fresh culture medium was added. After 15 h, images of wounds were captured with a Nikon Eclipse Ti-S microscope (Tokyo, Japan) with a 10× objective. The average width of wounds at different intervals was determined after cell migration, and the distance between wound edges was calculated. Wound closure area was measured using ImageJ software.

Cell migration and invasion assay

The membrane of Transwell inserts (3421, Corning) was coated with 0.25 mg/ml fibronectin (Sigma-Aldrich, F2006) or 1 mg/ml Matrigel (354248, Corning). Then, 5 × 10^4 cells were seeded on the insert and incubated for 48 h. Cells on the upper membrane that had not migrated or invaded were removed with cotton swabs. Migrating and invading cells were stained and fixed with 0.5% crystal violet, 3.7% formaldehyde, and 30% ethanol for 2 h. The central portion of the membrane was imaged on a Nikon Eclipse Ti-S microscope with an Intensilight C-HGFI illuminator and 10× objective. Migrating and invading cells were manually counted from four sites in each image.

Cell proliferation assay using CCK-8

A total of 1 × 10^3 cells per well were seeded in 96-well plates. After 24 h, cells were treated with drugs and incubated for 72 h. Cell viability was assessed using the EZ-Cytox Cell Viability Assay kit (EZ-3000, DoGen, Seoul, Korea), and absorbance (450 nm) was measured using a microplate reader (Tecan, CA).

scATAC-seq data processing and analysis

Cell ranger-atac (v1.2.0) demultiplex raw Base Call files were generated by Illumina sequencers and converted into FASTQ files using the cellranger mkfastq module. Chromium scATAC libraries include paired-end read 1 (containing the 50-bp insert) and read 2 (50-bp insert), the 10× barcode in the i5 index (16 bp) read, and the sample index in the i7 index (8 bp) read. FASTQ files were aligned to the

human reference genome (hg19) using the cellranger-atac count module, and the web summary, peak count matrix, and transcription factor (TF) count matrix were generated. Quality control was conducted using fraction reads in cells, the mapping quality, insert sizes, and the transcription start site (TSS) profile in the web summary following the 10× Genomics recommendation. The number of reads per cell exceeded 50,000 to saturate coverage. The peak count matrix and bam files were used as input for downstream analyses identifying cell clusters and differentially accessible regions (DARs).

Data integration, dimensionality reduction, clustering, and DAR analysis in scATAC-seq

The scATAC-seq datasets of LR cells produced 7,753 filtered nucleus barcodes. Overall scATAC-seq analysis was performed using the SnapATAC package (v.1.0.0)[2], which consists of snaptools for preprocessing and SnapATAC for clustering, annotation, motif discovery, and downstream analysis. First, a snap (single-nucleus accessibility profile) file was generated from bam files from cellranger-atac using snaptools (*https://github.com/r3fang/SnapATAC/wiki/FAQs*). This step included the addition of the cell-by-bin matrix to the snap file. The bin size was set to 5 kb following the recommendation of snaptools. Second, the snap file was loaded with snaptools, and our scATAC-seq dataset was integrated for batch effect correction using the harmony module in SnapATAC. Third, for high-quality barcodes, filtering was performed using the default parameters in SnapATAC, except that the mitochondrial genome was not included. The rest of the process was carried out according to the SnapATAC pipeline. For integration of the scRNA-seq and scATAC-seq datasets, a cell-by-gene matrix (gene activity score) was added based on the gene body region after conversion to a Seurat object using the 'snapToSeurat' module in SnapATAC. Subsequent processing was based on the 'scATAC-seq and scRNA-seq integration' section of Seurat. Using the 'TransferData' module in Seurat, pseudo-multiomics were created for each cell of scATAC-seq. Cells with a prediction score less than 0.3 were discarded. Significantly enriched peaks for each cluster were distinguished with the runMACS module[3] in SnapATAC, and a narrow peak and bedgraph were generated for each cluster. DARs were analyzed using the 'findDAR' module with the cutoff set to a P value of less than 0.05 and a Log_2(fold change) of greater than 0. For the interaction score, the 'predictGenePeakPair' module was used and the association between open chromatin regions (OCRs), and gene expression within ±250 kb centered on the TSS was calculated as Log_{10}(P value). Motif analysis for DARs was performed using HOMER (v4.11.1)[4] by employing the runHomer module in SnapATAC. Motifs were

analyzed based on 392 TFs in HOMER. pyGenomeTracks (v.3.5.1)[5] was used to summarize scATAC-seq with a genome browser.

Colony-forming assay

Cells were seeded in a 6-well plate (3506, Corning) at 1×10^3 cells/well and treated with 5fdC for 10 days. Colonies were fixed and stained with 0.5% crystal violet (3886, Sigma-Aldrich), 3.7% formaldehyde (F8775, Sigma-Aldrich), and 30% ethanol (100983, Merck Millipore). Colonies were counted using ImageJ software.

Flow cytometry–based apoptosis assay

Cells were seeded in 100-mm culture dishes at 1.5×10^6 per dish prior to experimental treatment. Apoptosis assays were performed using the FITC Annexin V Apoptosis Detection kit I (556547, BD Biosciences, NJ). Cells were analyzed using a BD FACSCalibur flow cytometer and CellQuest Pro software (BD Biosciences).

Immunofluorescence microscopy

H3122 cells and LR cells (2×10^2) were seeded in 8-well plates (15446, Lab-Tek II, NY), washed twice with ice-cold Dulbecco's Phosphate Buffered Saline (DPBS; LB001-02, Welgene), and fixed with 4% paraformaldehyde (P2031, Biosesang, Seongnam, Korea) for 2 h at room temperature. After three washes with ice-cold DPBS, cells were permeabilized for 10 min in 0.25% Triton X-100 (T9284, Sigma-Aldrich). After washing three times, cells were blocked overnight at 4°C in a humidified chamber. Cells were incubated with anti-Ki-67 (1:100, AB9260, Merck Millipore) and anti-γ-H2AX (1:500, 05-636, Merck Millipore) overnight at 4°C in a humidified chamber. After washing three times, cells were incubated with appropriate secondary antibodies conjugated to Alex488 Alexa488 or Alexa647 (1:200, A11011, A11034, Life Technologies, CA). Nuclei were stained with 4′,6-diamidino-2-phenylindole dihydrochloride (DAPI; D8417, Sigma-Aldrich). Images were captured with a laser-scanning microscope (Model LSM 800; ZEISS, Oberkochen, Germany).

Supplementary Fig. 1

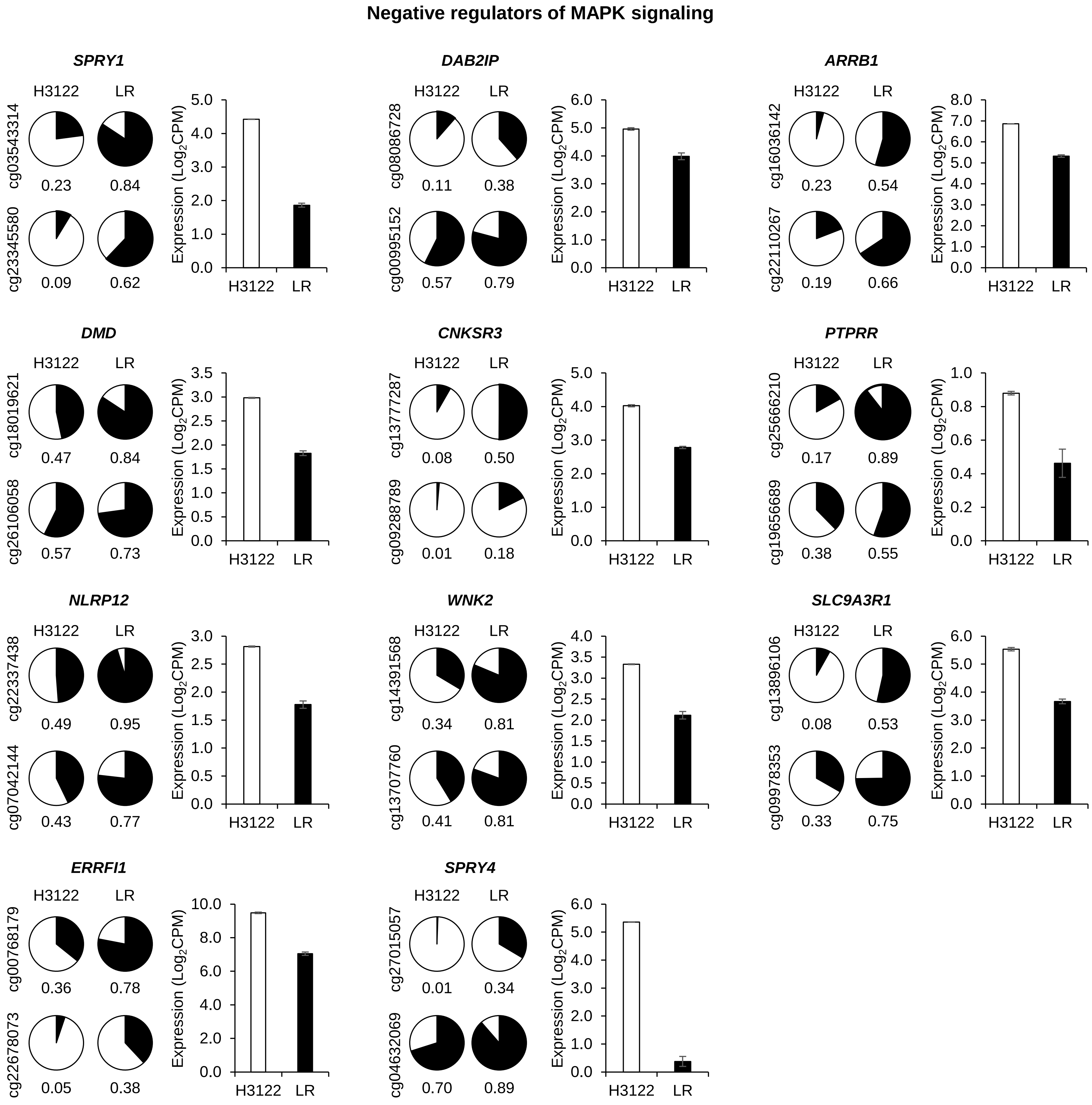

Supplementary Fig. 2. scRNA-seq analysis of H3122 and LR.

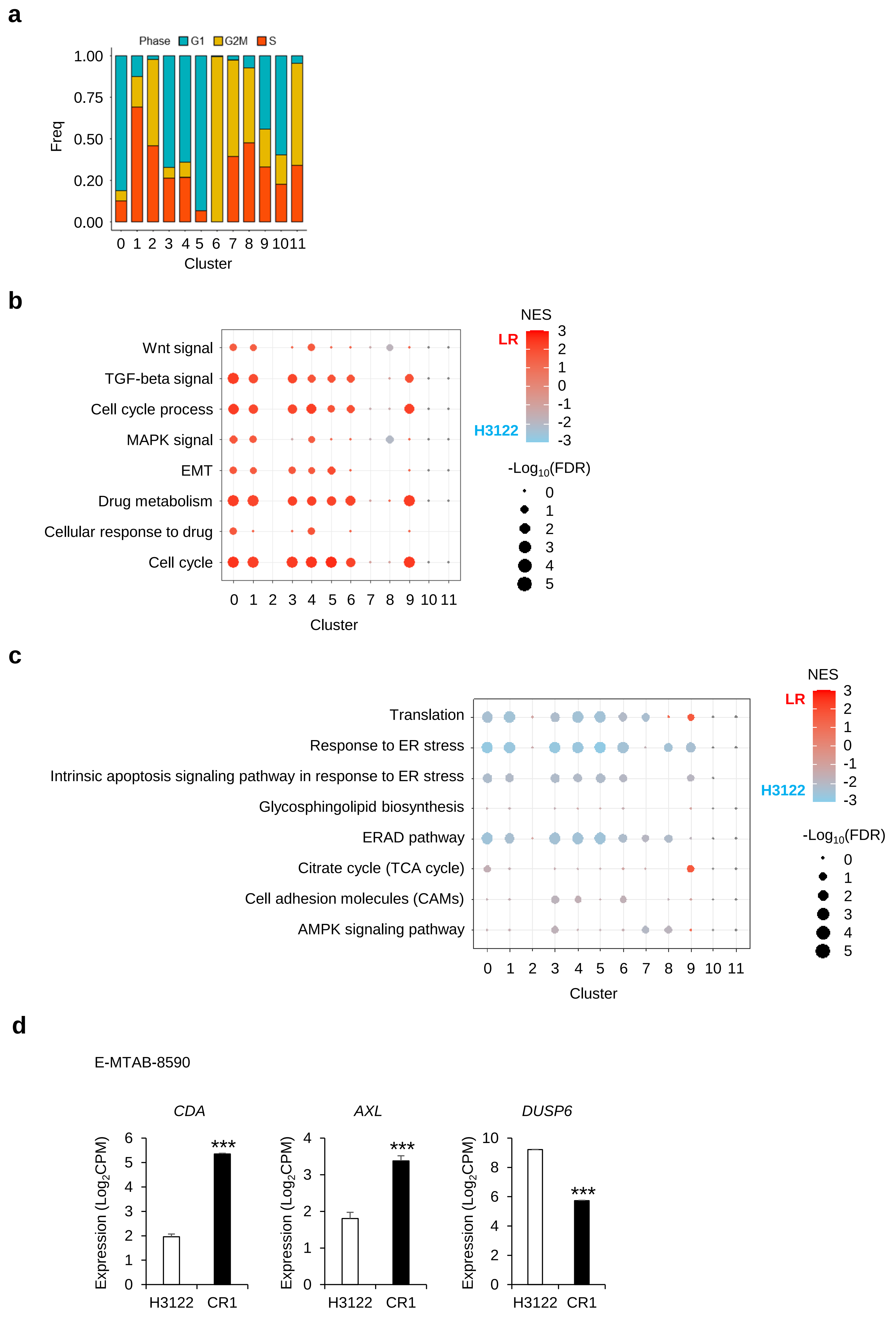

Supplementary Fig. 3. Cis regulatory elements controlling *CDA* expression in LR cells.

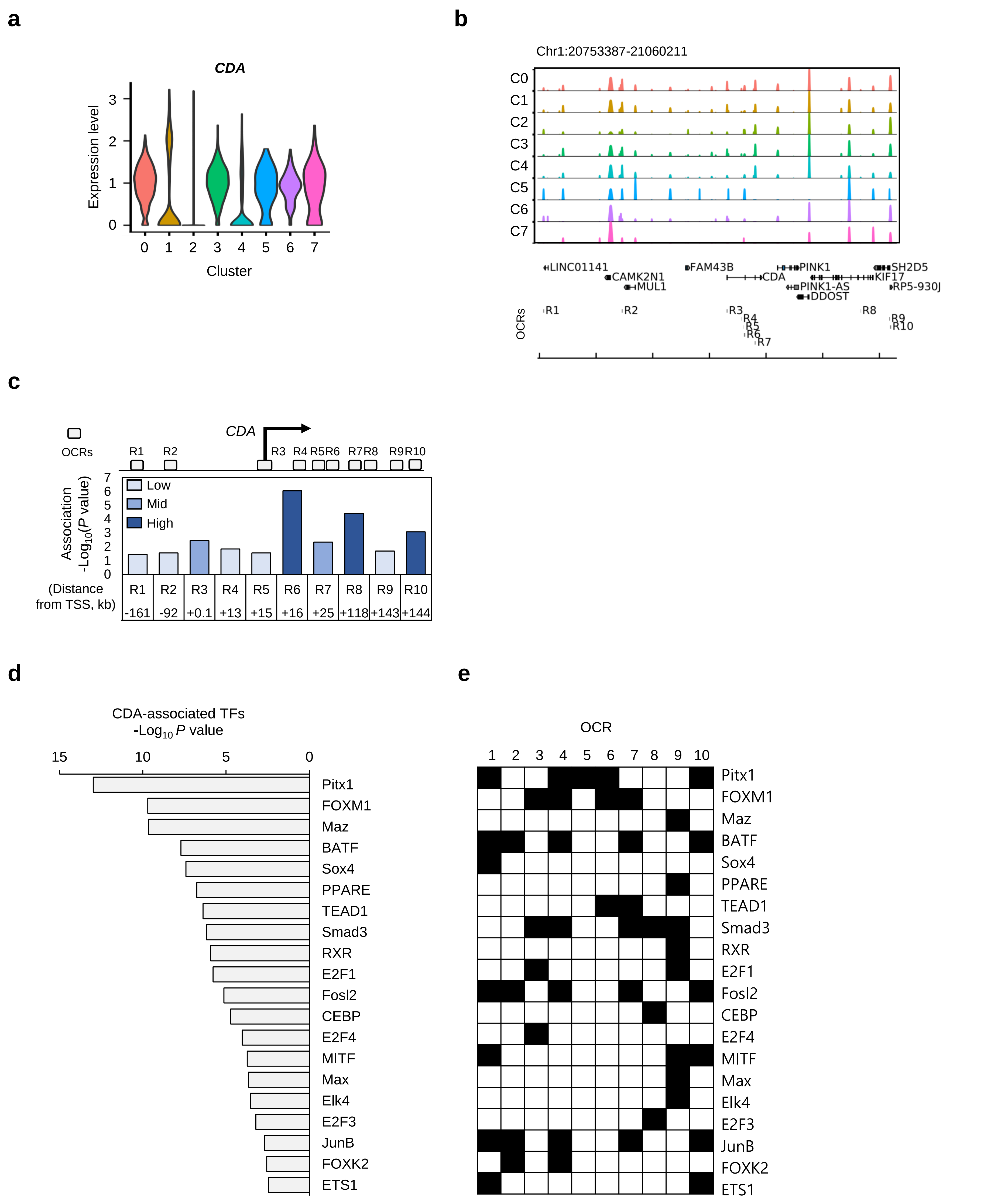

SUPPLEMENTARY TABLES

Supplementary Table 1. Primer sequences for qRT-PCR.

No.	Gene		Primer sequence (5' - 3')
1	β-Actin	Forward	CAAGAGATGGCCACGGCTGCT
		Reverse	TCCTTCTGCATCCTGTCGGCA
2	DNMT1	Forward	TATGGAAGGCTCGAGTGGGA
		Reverse	GTCCAGGATGTTGCCGAAGA
3	DNMT3A	Forward	CTTTTGCGTGGAGTGTGTGG
		Reverse	GCTTCCTCTTCTCAGCTGGG
4	DNMT3B	Forward	CCAACAACACGCAACCAGAG
		Reverse	CTGCCACAAGACAAACAGCC
5	TET1	Forward	AGGGCAGTGGAAAAGAAACCT
		Reverse	AGATGCCTCTTTCACTGGCTT
6	TET2	Forward	GTCAGCCCCATCACGTACAA
		Reverse	TTGGGCCGTCTCATGTATGG
7	TET3	Forward	CCGGAGAAGATCAAGCAGGA
		Reverse	CATGCTGTAAGGGTCGGAGG
8	DUSP6	Forward	GTCGATGAACGATGCCTATG
		Reverse	GATTGCAGAGAGTCCACCTG
9	AXL	Forward	GCTGCCTGTGTCCTCATCTT
		Reverse	CAGCTCTTCACTGATGCCCA
10	CDA	Forward	TGCCCCTACAGTCACTTTCC
		Reverse	CCAGTTGGTGCCAAACTCTC

Supplementary Table 2. 10× Genomics web summaries for scRNA-seq.

Quality index (10x Chromium)	H3122	LR
Number of Reads	309501554	298153253

Estimated Number of Cells	4981	4953
Mean Reads per Cell	62136	60196
Median Genes per Cell	3322	2187
Total Genes Detected	20561	20232
Fraction Reads in Cells	91.20%	55.30%
Valid Barcodes	98.50%	97.30%
Reads Mapped to Genome	93.00%	97.20%
Q30 Bases in Barcode	96.90%	97.50%
Q30 Bases in RNA Read	73.80%	93.10%
Q30 Bases in Sample Index	96.80%	97.40%
Q30 Bases in UMI	96.80%	97.40%

Supplementary Table 3. Primer sequences for bisulfite sequencing.

No.	Probe ID		Primer sequence (5' - 3')
1	cg04087271	Forward	TTTAGTGAAGGAGAGAAAAAGTG
		Reverse	CCTCCCATAATTTAAACTCAACA
2	cg20619374	Forward	AGGTAGGAAGGGGAGGTTATA
		Reverse	TCACCACCCTAACCAACACTA
3	cg06984156	Forward	TATAGATATTTGTGTTGAAGG
		Reverse	ATAACACATCACATCTTTACCAC

Supplementary Table 4. siRNA sequences for target genes.

No.	Gene	siRNA	siRNA sequence (5' - 3')
1		siRNA-1	GGAUUUCAGGGCAAUUGCU
2	CDA	siRNA-2	CCGAGCAACUUUUCUAAUU
3		siRNA-3	UGAGAGAGUUUGGCACCAA
4	FOXM1	siRNA-1	CUCUGACGCUUGUGACAGU

5	SMAD3	siRNA-1	GAGAGAUUCAACUUUCCAA
6	TEAD1	siRNA-1	AGUUUCCAGCUGGGAUCAA

Supplementary Table 5. 10x Genomics web summary for scATAC-seq.

Quality index (10x Chromium)	LR
Total number of read pairs	437456460
Estimated number of cells	7753
Mean reads per cell	56424
Median fragments per cell	6821
Q30 Bases in Read1	20394
Q30 Bases in Read2	95.74%
Q30 Bases in Barcode	93.62%
Q30 Bases in Sample Index	94.91%
Fraction of total read pairs mapped confidently to genome	97.30%
Fraction of fragments overlapping any targeted region (%)	85.18%

Supplementary Table 6. Differentially expressed genes in CDA-overexpressing cells (also see Fig. 2).

Gene	p_val	avg_logFC	pct.1	pct.2	p_val_adj
CDA	4.69E-52	1.195895198	1	0.076	8.45E-48
VIM	4.63E-50	0.832161526	0.412	0.011	8.35E-46
ZBED2	9.19E-35	0.262593928	0.235	0.005	1.66E-30
KRT81	2.86E-15	1.020678336	0.765	0.145	5.16E-11
COL5A1	3.55E-11	0.64094703	0.647	0.135	6.39E-07
COL4A2	5.17E-11	0.69570285	0.529	0.091	9.31E-07
S100A10	2.27E-09	1.125891111	1	0.995	4.10E-05
LGALS1	7.84E-09	1.272684634	1	0.956	0.000141266
CST6	1.02E-08	1.219997182	0.882	0.509	0.000184392
SH3BGRL3	1.96E-08	0.691906318	1	0.952	0.000352322
NEURL1B	6.48E-08	0.316992503	0.412	0.073	0.001167183
EPHB2	7.39E-08	0.305245659	0.824	0.314	0.001331678
IL32	2.11E-07	0.633567967	0.941	0.492	0.003794519
PDLIM4	1.62E-06	0.518846512	0.824	0.477	0.029270252
PDLIM7	2.33E-06	0.684995763	1	0.694	0.042029478
FSTL3	2.37E-06	0.315577937	0.588	0.196	0.042619088
GDF15	7.61E-06	-1.086414001	0.824	0.965	0.13712525
CD44	9.59E-06	0.290678903	0.647	0.236	0.172693723
PLP2	9.70E-06	0.448052902	1	0.687	0.174664738
AP3S1	1.29E-05	0.454684865	1	0.708	0.232132568
TAX1BP3	1.31E-05	0.370898761	0.882	0.555	0.236049437
PALLD	1.66E-05	0.362388779	0.588	0.202	0.299862557
BPIFA2	1.80E-05	-0.880999321	0.294	0.77	0.324591423
CCPG1	2.04E-05	-0.450710722	0.588	0.87	0.366774639
CALM1	2.42E-05	0.432013298	1	0.931	0.435371808
TKT	2.42E-05	-0.58324065	1	0.981	0.435490369
BPIFB1	2.58E-05	-1.598773403	0.118	0.639	0.464643111
TPM1	2.72E-05	1.052675832	0.765	0.421	0.490338303
C16orf74	3.20E-05	0.291897875	0.588	0.223	0.575869919
BASP1	3.31E-05	0.509123795	0.941	0.58	0.595497061
LDHB	4.27E-05	-0.388677914	1	0.987	0.769462288
PMEPA1	4.66E-05	0.854306734	0.882	0.767	0.838886474
FSCN1	4.98E-05	0.299076356	0.706	0.328	0.897161146

Supplementary Table 7. Transcription factors predicted to bind to the regulatory region of *CDA* (also see Fig. 4).

	gene	target	corr	interaction	gene2	corr_scRNA_LR	corr_scRNA_LRH3122	type
320024	chr1:20901755-20902366_CDA	TEAD1	0.150925177	1.332872029	CDA	0.102416771	0.032903403	enhancer
319992	chr1:20901755-20902366_CDA	E2F3	0.150925177	1.332872029	CDA	0.069426341	0.086600241	enhancer
319998	chr1:20901755-20902366_CDA	ETS1	0.150925177	1.332872029	CDA	0.059228419	0.055514122	enhancer
319996	chr1:20901755-20902366_CDA	Elf4	0.150925177	1.332872029	CDA	0.058174909	0.109124049	enhancer
320001	chr1:20901755-20902366_CDA	ETV4	0.150925177	1.332872029	CDA	0.049015012	-0.078706703	enhancer
320026	chr1:20901755-20902366_CDA	Tgif2	0.150925177	1.332872029	CDA	0.047908282	-0.003861792	enhancer
320000	chr1:20901755-20902366_CDA	Etv2	0.150925177	1.332872029	CDA	0.046240777	-0.000191734	enhancer
320025	chr1:20901755-20902366_CDA	Tgif1	0.150925177	1.332872029	CDA	0.032613641	-0.153583634	enhancer
319993	chr1:20901755-20902366_CDA	E2F6	0.150925177	1.332872029	CDA	0.028365154	-0.01517582	enhancer
320016	chr1:20901755-20902366_CDA	Nanog	0.150925177	1.332872029	CDA	0.02282812	-0.018742921	enhancer
320003	chr1:20901755-20902366_CDA	GABPA	0.150925177	1.332872029	CDA	0.019944225	-0.022472268	enhancer
320027	chr1:20901755-20902366_CDA	ZFX	0.150925177	1.332872029	CDA	0.018830529	0.02169422	enhancer
320023	chr1:20901755-20902366_CDA	Tbx6	0.150925177	1.332872029	CDA	0.017125889	0.030701902	enhancer
320015	chr1:20901755-20902366_CDA	Meis1	0.150925177	1.332872029	CDA	0.0146181	0.01063325	enhancer
320004	chr1:20901755-20902366_CDA	HIC1	0.150925177	1.332872029	CDA	0.009115437	-0.02364584	enhancer
320014	chr1:20901755-20902366_CDA	KLF14	0.150925177	1.332872029	CDA	0.007105143	0.019523784	enhancer
320030	chr1:20901755-20902366_CDA	ZNF711	0.150925177	1.332872029	CDA	0.006049427	-0.02350004	enhancer
319994	chr1:20901755-20902366_CDA	EBF1	0.150925177	1.332872029	CDA	0.005480371	0.019919692	enhancer
320010	chr1:20901755-20902366_CDA	Hoxd11	0.150925177	1.332872029	CDA	0.002211628	-0.002793434	enhancer
320052	chr1:20915290-20915856_CDA	Smad3	0.251775991	2.525345901	CDA	0.10057137	-0.119507549	enhancer
320034	chr1:20915290-20915856_CDA	E2F1	0.251775991	2.525345901	CDA	0.096998282	0.200647588	enhancer
320035	chr1:20915290-20915856_CDA	E2F4	0.251775991	2.525345901	CDA	0.079169656	0.114594325	enhancer
320033	chr1:20915290-20915856_CDA	Bcl11a	0.251775991	2.525345901	CDA	0.058596189	0.033945242	enhancer
320053	chr1:20915290-20915856_CDA	Smad4	0.251775991	2.525345901	CDA	0.050332579	-0.029921995	enhancer
320057	chr1:20915290-20915856_CDA	Znf263	0.251775991	2.525345901	CDA	0.041232037	0.035036421	enhancer
320051	chr1:20915290-20915856_CDA	Smad2	0.251775991	2.525345901	CDA	0.039503168	-0.006329958	enhancer
320054	chr1:20915290-20915856_CDA	Sp2	0.251775991	2.525345901	CDA	0.037973393	0.041730416	enhancer
320055	chr1:20915290-20915856_CDA	Sp5	0.251775991	2.525345901	CDA	0.037482646	0.041372461	enhancer
320043	chr1:20915290-20915856_CDA	KLF6	0.251775991	2.525345901	CDA	0.036127395	0.11024855	enhancer
320042	chr1:20915290-20915856_CDA	KLF5	0.251775991	2.525345901	CDA	0.035043624	-0.108680119	enhancer
320036	chr1:20915290-20915856_CDA	E2F6	0.251775991	2.525345901	CDA	0.028365154	-0.01517582	enhancer
320038	chr1:20915290-20915856_CDA	Foxo1	0.251775991	2.525345901	CDA	0.017405873	-0.020080355	enhancer
320041	chr1:20915290-20915856_CDA	Klf4	0.251775991	2.525345901	CDA	0.013325394	-0.171649919	enhancer
320059	chr1:20915290-20915856_CDA	ZNF467	0.251775991	2.525345901	CDA	0.00789237	-0.177693737	enhancer
320040	chr1:20915290-20915856_CDA	KLF14	0.251775991	2.525345901	CDA	0.007105143	0.019523784	enhancer
320058	chr1:20915290-20915856_CDA	ZNF416	0.251775991	2.525345901	CDA	0.005156093	0.014698395	enhancer
320094	chr1:20928134-20928356_CDA	Pitx1	0.614300094	2.02668942	CDA	0.149919651	0.009663815	enhancer
320076	chr1:20928134-20928356_CDA	FOXM1	0.614300094	2.02668942	CDA	0.128503671	0.3057048	enhancer
320061	chr1:20928134-20928356_CDA	BATF	0.614300094	2.02668942	CDA	0.113495139	0.356246086	enhancer
320098	chr1:20928134-20928356_CDA	Smad3	0.614300094	2.02668942	CDA	0.10057137	-0.119507549	enhancer
320066	chr1:20928134-20928356_CDA	Fosl2	0.614300094	2.02668942	CDA	0.090712368	0.171258673	enhancer
320088	chr1:20928134-20928356_CDA	JunB	0.614300094	2.02668942	CDA	0.062344434	-0.057970673	enhancer
320074	chr1:20928134-20928356_CDA	FOXK2	0.614300094	2.02668942	CDA	0.060659477	-0.068415492	enhancer
320068	chr1:20928134-20928356_CDA	FOXA1	0.614300094	2.02668942	CDA	0.055202984	-0.070047765	enhancer
320069	chr1:20928134-20928356_CDA	FOXA1	0.614300094	2.02668942	CDA	0.055202984	-0.070047765	enhancer
320073	chr1:20928134-20928356_CDA	FOXK1	0.614300094	2.02668942	CDA	0.048253054	-0.034154374	enhancer
320062	chr1:20928134-20928356_CDA	DLX1	0.614300094	2.02668942	CDA	0.042887334	0.048515434	enhancer
320103	chr1:20928134-20928356_CDA	TRPS1	0.614300094	2.02668942	CDA	0.035456658	-0.047066171	enhancer
320063	chr1:20928134-20928356_CDA	DLX2	0.614300094	2.02668942	CDA	0.032294213	0.010898198	enhancer
320079	chr1:20928134-20928356_CDA	GATA3	0.614300094	2.02668942	CDA	0.025770082	-0.023941971	enhancer
320090	chr1:20928134-20928356_CDA	Nanog	0.614300094	2.02668942	CDA	0.02282812	-0.018742921	enhancer
320080	chr1:20928134-20928356_CDA	GSC	0.614300094	2.02668942	CDA	0.022800733	-0.07528887	enhancer
320070	chr1:20928134-20928356_CDA	Foxa2	0.614300094	2.02668942	CDA	0.021247738	-0.064217969	enhancer
320065	chr1:20928134-20928356_CDA	Fos	0.614300094	2.02668942	CDA	0.01362663	-0.04832221	enhancer
320075	chr1:20928134-20928356_CDA	FoxL2	0.614300094	2.02668942	CDA	0.00515646	-0.026926229	enhancer
320085	chr1:20928134-20928356_CDA	Hoxd11	0.614300094	2.02668942	CDA	0.002211628	-0.002793434	enhancer
320170	chr1:20930570-20931409_CDA	Six2	-0.215457485	7.751949758	CDA	-0.001341772	0.014083809	repressor
320179	chr1:20930570-20931409_CDA	ZEB2	-0.215457485	7.751949758	CDA	-0.007812725	0.017316704	repressor
320178	chr1:20930570-20931409_CDA	ZEB1	-0.215457485	7.751949758	CDA	-0.010290529	0.002500635	repressor
323070	chr1:21033382-21033587_CDA	Sox21	-0.250865574	2.691636245	CDA	-0.002041352	-0.018282902	repressor
323067	chr1:21033382-21033587_CDA	RARa	-0.250865574	2.691636245	CDA	-0.008480348	-0.073119399	repressor
323241	chr1:21059311-21060211_CDA	Pitx1	0.209629015	3.189348049	CDA	0.149919651	0.009663815	enhancer
323199	chr1:21059311-21060211_CDA	BATF	0.209629015	3.189348049	CDA	0.113495139	0.356246086	enhancer
323221	chr1:21059311-21060211_CDA	Fosl2	0.209629015	3.189348049	CDA	0.090712368	0.171258673	enhancer
323235	chr1:21059311-21060211_CDA	MITF	0.209629015	3.189348049	CDA	0.075733465	0.063905574	enhancer
323233	chr1:21059311-21060211_CDA	Max	0.209629015	3.189348049	CDA	0.074786615	-0.079190638	enhancer
323209	chr1:21059311-21060211_CDA	Elk4	0.209629015	3.189348049	CDA	0.073467562	0.010467266	enhancer
323230	chr1:21059311-21060211_CDA	JunB	0.209629015	3.189348049	CDA	0.062344434	-0.057970673	enhancer
323213	chr1:21059311-21060211_CDA	ETS1	0.209629015	3.189348049	CDA	0.059228419	0.055514122	enhancer
323207	chr1:21059311-21060211_CDA	Elf4	0.209629015	3.189348049	CDA	0.058174909	0.109124049	enhancer
323216	chr1:21059311-21060211_CDA	ETV4	0.209629015	3.189348049	CDA	0.049015012	-0.078706703	enhancer
323248	chr1:21059311-21060211_CDA	Tgif2	0.209629015	3.189348049	CDA	0.047908282	-0.003861792	enhancer
323215	chr1:21059311-21060211_CDA	Etv2	0.209629015	3.189348049	CDA	0.046240777	-0.000191734	enhancer
323206	chr1:21059311-21060211_CDA	ELF1	0.209629015	3.189348049	CDA	0.037839022	-0.011882705	enhancer
323236	chr1:21059311-21060211_CDA	MNT	0.209629015	3.189348049	CDA	0.035325756	-0.074115379	enhancer
323246	chr1:21059311-21060211_CDA	TEAD3	0.209629015	3.189348049	CDA	0.035150565	0.066431526	enhancer
323205	chr1:21059311-21060211_CDA	CLOCK	0.209629015	3.189348049	CDA	0.034614134	0.007762054	enhancer
323208	chr1:21059311-21060211_CDA	Elk1	0.209629015	3.189348049	CDA	0.032898712	0.060725872	enhancer
323247	chr1:21059311-21060211_CDA	Tgif1	0.209629015	3.189348049	CDA	0.032613641	-0.153583634	enhancer
323200	chr1:21059311-21060211_CDA	bHLHE40	0.209629015	3.189348049	CDA	0.02804646	-0.01769445	enhancer
323224	chr1:21059311-21060211_CDA	GABPA	0.209629015	3.189348049	CDA	0.019944225	-0.022472268	enhancer
323201	chr1:21059311-21060211_CDA	bHLHE41	0.209629015	3.189348049	CDA	0.018658587	0.037334189	enhancer

323240	chr1:21059311-21060211_CDA	NPAS2	0.209629015	3.189348049	CDA	0.015985607	-0.198206016	enhancer
323234	chr1:21059311-21060211_CDA	Meis1	0.209629015	3.189348049	CDA	0.0146181	0.01063325	enhancer
323220	chr1:21059311-21060211_CDA	Fos	0.209629015	3.189348049	CDA	0.01362663	-0.04832221	enhancer
323242	chr1:21059311-21060211_CDA	PRDM1	0.209629015	3.189348049	CDA	0.007037706	0.044942365	enhancer
323249	chr1:21059311-21060211_CDA	USF1	0.209629015	3.189348049	CDA	0.006986314	0.047261717	enhancer
323228	chr1:21059311-21060211_CDA	Hoxd11	0.209629015	3.189348049	CDA	0.002211628	-0.002793434	enhancer

Supplementary Table 8. Overview of primary cancer cells from NSCLC patients with ALK rearrangements (also see Fig. 6d).

Patient No.	Remark	Sex	Age (yrs)	Diagnosis	Specimen type
SNU-2535	ALK+, Crizotinib-resistant G1269A	Female	57	NSCLC (ADC)-ALK(+)	Pleural effusion
SNU-2550	ALK+, Crizotinib-resistant , S106F	Female	35	NSCLC (ADC)-ALK(+)	Pleural effusion
SNU-2563	ALK+, Crizotinib-resistant	Female	49	NSCLC (ADC)-ALK(+)	Pleural effusion
SNU-3166	Crizotinib-naïve ALK+ NSCLC	Female	33	NSCLC (ADC)-ALK(+)	Ascites

Supplementary Table 9. Upregulation of CDA in cancer cell lines resistant to anticancer drugs.

Cell line	Cancer type	Anticancer drug	Increase in CDA expression (resistant/parental)	Reference
MDA-MB-231	Breast cancer	Palbociclib	2.95	6
HCT116	Colorectal cancer	Trametinib	2.27	7
PEO1	Ovarian cancer	Olaparib	1.87	8
PC9	Non-small-cell lung cancer	Gefitinib	1.46	9

REFERENCES

1. Stuart, T. *et al.* Comprehensive Integration of Single-Cell Data. *Cell* **177,** 1888-1902 e1821 (2019).
2. Fang, R. *et al.* Comprehensive analysis of single cell ATAC-seq data with SnapATAC. *Nat. Commun.* **12,** 1337 (2021).
3. Zhang, Y. *et al.* Model-based analysis of ChIP-Seq (MACS). *Genome Biol.* **9,** R137 (2008).
4. Heinz, S. *et al.* Simple combinations of lineage-determining transcription factors prime cis-regulatory elements required for macrophage and B cell identities. *Mol. Cell* **38,** 576-589 (2010).
5. Lopez-Delisle, L. *et al.* pyGenomeTracks: reproducible plots for multivariate genomic datasets. *Bioinformatics* **37,** 422-423 (2021).
6. Lypova, N. *et al*. Targeting Palbociclib-Resistant Estrogen Receptor-Positive Breast Cancer Cells via Oncolytic Virotherapy. *Cancers (Basel)* **11,** (2019).
7. Wagner, S. *et al*. Suppression of interferon gene expression overcomes resistance to MEK inhibition in KRAS-mutant colorectal cancer. *Oncogene* **38,** 1717-1733 (2019).
8. Yamamoto, T.M. *et al*. Activation of Wnt signaling promotes olaparib resistant ovarian cancer. *Mol. Carcinog.* **58,** 1770-1782 (2019).
9. Song, Y.A. *et al*. Apatinib preferentially inhibits PC9 gefitinib-resistant cancer cells by inducing cell cycle arrest and inhibiting VEGFR signaling pathway. *Cancer Cell Int.* **19,** 177 (2019).

Supplementary Fig. Legends

Supplementary Fig. 1 DNA methylation and mRNA expression of negative regulators of MAPK signaling in H3122 and LR. Left: DNA methylation levels plotted as pie charts representing the percentage of methylation (black) detected for individual Infinium Human Methylation 450K BeadChip probes. Right: mRNA levels from RNA-seq.

Supplementary Fig. 2 scRNA-seq analysis of H3122 and LR. a Frequency of cell-cycle phases in each cluster. **b, c** Enriched Gene Ontology terms for genes upregulated in LR versus H3122. NES is normalized enrichment score; circle size represents false discovery rate as $-\mathrm{Log}_{10}$(FDR). **d** Expression of *CDA*, *AXL*, and *DUSP6* in H3122 cells and crizotinib-resistant H3122 (CR1) cells. RNA-seq data were obtained from the European Nucleotide Archive (E-MTAB-8590).

Supplementary Fig. 3 Cis regulatory elements controlling *CDA* expression in LR cells. a Violin plot of *CDA* expression in each cluster (Fig. 4a). **b** ATAC signal of each cluster within ±250 kb of the *CDA* transcription start site (TSS). **c** Association of OCRs (R1-R10) with *CDA* expression. Distance from the *CDA* TSS is shown. Association of OCRs is classified as high ($P < 0.001$), mid ($P = 0.001$-0.01), and low ($P = 0.01$-0.05). **d** Top 20 TFs predicted to bind to OCRs of *CDA*. **e** Black squares indicate TF-binding OCRs.
